# Multimodal in-vehicle lighting system increases daytime light exposure and alertness in truck drivers under Arctic winter conditions

**DOI:** 10.1038/s41598-024-60308-y

**Published:** 2024-04-30

**Authors:** Roland F. J. Popp, Julia Ottersbach, Thomas C. Wetter, Sebastian Schüler, Siegfried Rothe, Daniel Betz, Siegmund Staggl, Markus Canazei

**Affiliations:** 1 Department of Psychiatry and Psychotherapy, Center of Sleep Medicine, University of Regensburg, 93053 Regensburg, Germany; 2 Institute of Experimental Psychology, University of Regensburg, 93053 Regensburg, Germany; 3 Mercedes-Benz AG, 71059 Sindelfingen, Germany; 4 Department of Psychology, University of Innsbruck, 6020 Innsbruck, Austria

**Keywords:** Psychology, Engineering

## Abstract

Drowsiness while driving negatively impacts road safety, especially in truck drivers. The present study investigated the feasibility and alerting effects of a daylight-supplementing in-truck lighting system (DS) providing short-wavelength enriched light before, during, and after driving. In a within-participants design, eight truck drivers drove a fully-loaded truck under wintry Scandinavian conditions (low daylight levels) with a DS or placebo system for five days. Subjective and objective measures of alertness were recorded several times daily, and evening melatonin levels were recorded three times per study condition. DS significantly increased daytime light exposure without causing negative side effects while driving. In addition, no negative carry-over effects were observed on evening melatonin and sleepiness levels or on nighttime sleep quality. Moreover, objective alertness (i.e., psychomotor vigilance) before and after driving was significantly improved by bright light exposure. This effect was accompanied by improved subjective alertness in the morning. This field study demonstrated that DS was able to increase daytime light exposure in low-daylight conditions and to improve alertness in truck drivers before and after driving (e.g., during driving rest periods). Further studies are warranted to investigate the effects of daylight-supplementing in-cabin lighting on driving performance and road safety measures.

## Introduction

Drowsiness while driving is a major risk factor for road safety and accounts for 15–20% of all traffic accidents in non-commercial drivers^1,2^ as well as in commercial truck drivers^3,4^. Reduced alertness due to drowsiness can cause severe traffic accidents during the transport of goods^5^, which has high risk for personal and property damage due to the weight of loaded vehicles and consequent increased stopping distances. Thus, even minor errors by truck drivers may cause major crashes with potentially fatal consequences.

Professional truck drivers are at especially high risk for excessive drowsiness while driving on monotonous highways, as they cover long distances using highly automated driving routines, are on the road for many hours at a time, and have prolonged work hours (not necessarily operating the vehicle). This may also result in task-related fatigue^6^. Monotony, often occurring during stretches of night driving and on highways, is a contributing factor to increased sleepiness while driving^7,8^. Other risk factors in transportation are related to irregular working hours and the prevalence of daytime sleepiness, poor sleep quality, obesity, and sleep disorders (e.g., sleep-related breathing disorders, which are often associated with increased body mass index), which are more common in truck drivers than in the general population^9–12^. Truck drivers have a high risk of sleepiness-induced crashes. In a survey of commercial long-haul truck drivers, one quarter of drivers reported that they had fallen asleep while driving at least once in the past year, and nearly half of all truck drivers reported to have fallen asleep while driving at least once in their career^12^. It is therefore crucial to reduce drowsiness due to sleepiness in truck drivers and support their alertness, especially under monotonous driving conditions.

Common countermeasures to combat sleepiness in drivers caused by sleep deprivation, poor sleep, or prolonged wakefulness, such as turning on the audio system or opening the windows, are mostly ineffective and provide short-term benefits at best^14^. The use of stimulants, such as caffeine or energy drinks, may also cause temporary alerting effects. However, these can have negative carry-over effects on nocturnal sleep and lead to habituation in the long term^15–17^. Other person-centered approaches aim at either supporting good sleep quality and quantity (e.g., by reducing overnight traffic noise along motorways^18^) or recommend exercising and napping during driving breaks^19^, which are not always feasible. Recent research has provided evidence of the effectiveness, feasibility, and relevance of various measures to counteract drowsiness or sleepiness for commercial motor vehicle operation^20^. One technological focus of research in the field of road safety is the automatic detection of driver drowsiness using assistance systems or the implementation of collision avoidance systems^20,21^. Other systems are obligatory for vehicles in the European Union starting in the summer of 2022^22^.

A new method to counteract sleepiness and promote alertness uses light supplementation. Research has shown that both bright polychromatic light and blue-enriched light can have acute alerting effects both during the night and day^23–25^. This acute alerting effect might act independently from circadian light effects^26^. Initial studies have shown that additional in-cabin lighting reduced drowsiness during nighttime driving in passenger cars^27,28^ as well as during dawn and twilight^29^. To date, no studies have objectively investigated the daytime alerting effects of supplementary short wavelength-enriched white light in truck drivers under naturalistic conditions.

As a proof of concept, we aimed to evaluate the feasibility and alerting effects of an in-vehicle lighting system. The system was designed to supplement the low level of daylight and increase the exposure of truck drivers to light before, during, and after driving. We simulated two consecutive workweeks with 8-h workdays for professional long-haul truck drivers in a wintry arctic environment with limited daylight. The drivers’ diurnal light exposure was continuously recorded, and a cabin light supplementation condition was compared with a placebo condition considering outdoor illuminance levels. The aim of the study was to determine the feasibility and efficiency of using a daylight-supplementing in-truck lighting system (DS) to supplement daylight for truck drivers while maintaining a high acceptance rate. We expected that DS would not have a negative impact on visual comfort while driving and working in the truck. Furthermore, we hypothesized that additional light, especially with a blue-enriched polychromatic spectrum, would affect alertness and counteract sleep-related drowsiness assessed using objective and subjective psychometric measures. Any effects of DS may be particularly pronounced in the Arctic winter, when outdoor light levels are low. As secondary study objectives, we assessed the prediction that DS would not have adverse effects on the sleep–wake system (i.e., no changes in evening melatonin level increase; no increased alertness before bedtime; no negative effects on sleep quality).

## Methods

### Study interventions

Two Mercedes-Benz Actros 1845 LS trucks (Daimler Truck AG, Leinfelden-Echterdingen, Germany) were equipped with a newly developed lighting and alleged air refreshing system. Drivers received unspecific information about the study purpose and were informed that they were testing newly designed in-cabin features (with regards to lighting and air refreshing) intended to improve driver fitness.

#### Daylight-supplementing in-cabin lighting system (DS)

**Figure 1:**
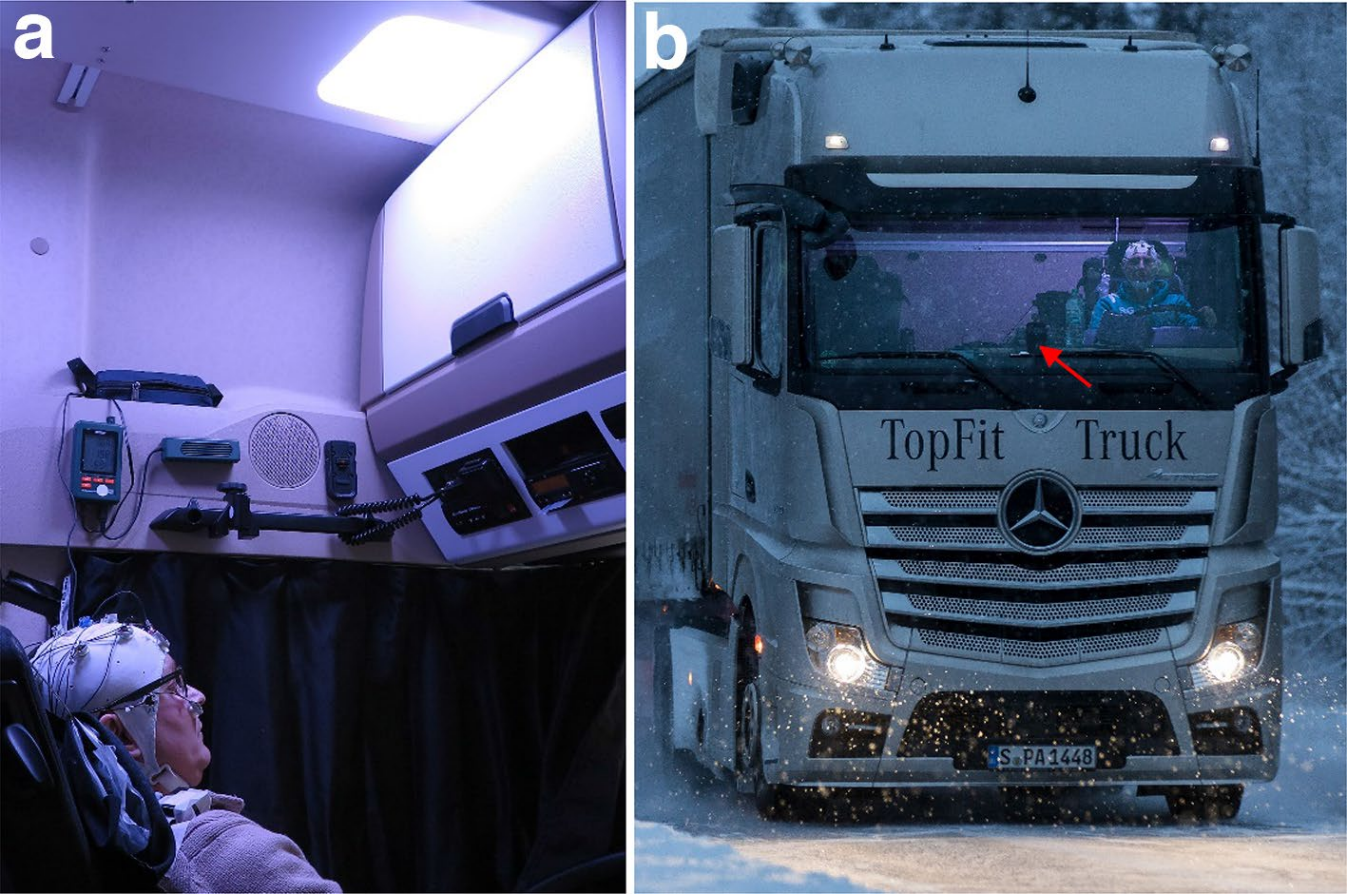
The daylight-supplementing in-cabin light system (DS) in its two usage modes; (**a**) blue-enriched light exposure before and after driving (DS++); a highly reflective, vertical surface increased the light level at the driver’s eyes; (**b**) daylight supplementation with blueish-white light while driving (DS+); red arrow indicates the position of the windshield photosensor, which continuously measured illuminances to control how much light should be supplemented by the DS.

A newly developed lighting system was mounted on the ceiling above the driver’s seat, which provided short wavelength-enhanced light during breaks in the morning and afternoon (referred to as DS^++^; Fig. 1a). The luminaire also dynamically supplemented daylight while driving (referred to as DS^+^; Fig. 1b) using a photosensor mounted behind the windshield (Supplementary Materials A.1 & A.2), which controlled the supplementation of light in the cabin based on the prevailing ambient illuminance levels measured by the photosensor.

#### Placebo condition

In the placebo condition, the lighting system in the cabin remained unchanged but provided no supplemented light; drivers were informed that they were now exposed to non-visible ultraviolet light (placebo UV) via the DS and ionized air provided by a system next to the driver’s seat (placebo AIR; Supplementary Materials A.3). These two components were used in the placebo condition to (i) allow a direct comparison of the DS cabin lighting system with a visual placebo (DS^+^ vs. placebo UV) and (ii) hide the fact that the study focused solely on the lighting intervention.

For the UV light dummy, a control switch built into the truck-driver’s cabin lit up during the placebo UV condition, but no supplemental visual or non-visual light was administered. The mockup air refreshing system consisted of a metal box with a control panel placed behind the co-driver’s seat, which circulated the air in the cabin and produced a humming noise (the system is depicted in Supplementary Materials A.3, Fig. A.3). Participants were told that the system was supposed to ionize and refresh the air within the cabin.

### Study protocol and assessments

The study was conducted on the roads around the Daimler Truck AG research facility near the Rovaniemi airport (Finland) and took place during the winter under naturally low daylight levels (November 21 to February 10). The present study mimicked two consecutive 5-day-working periods of truck drivers under naturalistic driving conditions. Following a counterbalanced and random order, professional truck drivers participated in the field study for five days under each condition (DS and placebo). The drivers drove a fully-loaded 40-t semitrailer daily on the same motorway route from Rovaniemi airport to Sodankylä and back again (about 260 km).

To increase monotony during the test drives, the radio of the truck was disabled. Truck drivers slept in their trucks for six consecutive nights in each study week (from Sunday to Saturday) and were instructed to enter their beds at 23:00 and to get up at 06:00 (using an alarm clock if necessary). They were asked to document their sleep onset and offset times and rate their subjective sleep quality after each of the five study nights (Monday to Friday) in both study conditions. Subjective sleep quality was further evaluated in the morning after each study night (Sunday to Saturday) using the Self-Assessment Scale for Sleeping and Awakening Quality (SSA). Using responses to the questionnaire, overall self-rated sleep quality, awakening quality, and somatic complaints during the night were quantified. Objective sleep quality was assessed using polysomnography (PSG) twice per study week (Monday and Friday). Table 1 gives an overview of the daily study protocol (see also Supplementary Materials B.7, Fig. B.2 for a representative depiction). A comprehensive overview of all applied measures and assessments is provided in Supplementary Materials B, accompanied by detailed descriptions.

**Table 1:** Study procedure.

Start time	Outcome measures/events	Light condition
Morning		
08:40	KSS—subjective sleepiness score	room lighting
08:45	10-min PVT – reaction speed and number of lapses (reactions after 500 ms)	room lighting
08:55	start recordings of wake EEG and light exposure at eye level (LuxBlick 2.0 device)	room lighting
09:00	start intervention: DS^++^/UV & AIR break in reclined driver’s seat	DS^++^ (*)/placebo
09:45	10-min PVT – reaction speed and number of lapses (reactions after 500 ms)	DS^++^ (*)/placebo
09:55	KSS—subjective sleepiness score	DS^++^ (*)/placebo
10:00	end DS^++^, start DS^+^/continue placebo UV, placebo AIR	DS^++^ (*)/placebo
Driving period		
10:00	truck driving start (at Rovaniemi airport)	DS^+^/placebo
12:00	KSS—subjective sleepiness score (5-min break at Sodankylä)	DS^+^/placebo
14:00	truck driving end (at Rovaniemi airport)	DS^+^/placebo
Afternoon		
14:00	end DS^+^, start DS^++^/continue UV& AIR	DS^++^/placebo
14:05	KSS—subjective sleepiness score	DS^++^/placebo
14:10	10-min PVT – reaction speed and number of lapses (reactions after 500 ms) break in reclined driver’s seat	DS^++^/placebo
15:15	10-min PVT – reaction speed and number of lapses (reactions after 500 ms)	DS^++^/placebo
15:30	KSS—subjective sleepiness score	DS^++^/placebo
15:40	end recordings of wake EEG and light exposure at eye level (LuxBlick 2.0 device)	DS^++^/placebo
15:45	end intervention	DS^++^/placebo
Late afternoon and evening period (KSS and saliva sampling: Monday, Wednesday, and Friday; PVT: Monday and Friday)		
17:00–22:00	Hourly KSS (subjective sleepiness) and saliva sample (melatonin level)	room lighting
22:30	10-min PVT – reaction speed and number of lapses (reactions after 500 ms)	room lighting
22:50	KSS (subjective sleepiness) and saliva sample (melatonin level)	room lighting

Note: room lighting at the study center comprised warm-white lighting (2700 Kelvin) with a mean illuminance of approximately 100 lx at eye level; * DS^++^ was not applied on Thursdays in the active study condition (instead, placebo light was used in the morning).

KSS, Karolinska Sleepiness Scale; PVT, psychomotor vigilance test; DS: active light condition with DS^+^, adaptive lighting during driving and DS^++^, bright light during morning and afternoon rests; UV, placebo UV lighting, AIR, placebo air refreshing in the placebo condition.

The truck drivers performed a test of objective alertness (10-min Psychomotor Vigilance Task [PVT]^30^) twice in the morning and twice in the afternoon under DS^++^ and the placebo condition. These tests took place in the driver’s seat, immediately before and after the daily driving periods (test times at 08:45 and 09:45 [morning] and at 14:05 and 15:10 [afternoon]). In addition to objective alertness measures, we assessed self-reported sleepiness during the day using the Karolinska Sleepiness Scale (KSS)^5^. KSS data were also recorded daily at 12:00 during a 5-min driving break in a motorway parking lot (near Sodankylä). The driving and testing phases required 8-h workdays in the cabin. To quantify potential negative carry-over effects of increased daytime light exposure on late evening sleepiness and sleep, we additionally measured evening melatonin levels and recorded sleepiness ratings in the evening.

On Mondays, Wednesdays, and Fridays, sleepiness in the evening was quantified using self-reported KSS ratings. Saliva melatonin samples (Blue Cap Salivette; Sarstedt AG & Co. KG, Nümbrecht, Germany) were collected seven times between 17:00 and 22:45. These samples were double-analyzed using Melatonin direct Saliva ELISA (RE54041; Tecan, Männedorf, Switzerland). Moreover, the PVT was performed immediately before the truck drivers went to bed in their trucks on Mondays and Fridays. At the end of the study week, on Saturday morning, participants filled in the acceptance questionnaire and were asked about aspects related to visual comfort and negative visual effects. The intervention components (DS^+^, placebo UV, and placebo AIR) were evaluated retrospectively with regards to the driving periods.

While driving, we also collected objective driving performance data (Fleetboard; Daimler Truck AG) and recorded wake electroencephalograms (EEGs) of the truck drivers (using alpha spindle rates as a marker for neurophysiological drowsiness). However, driving data and spindle rates were not subjected to data analysis due to insufficient data quality and are not reported.

### Study participants

Professional truck drivers were recruited by the road-testing department of Daimler Truck AG in Woerth (Germany) and paid for their participation. The recruitment process was biased towards men, as the vast majority of professional truck drivers are male. Inclusion criteria for participation were regular bedtimes and willingness to comply with study protocols (e.g., to refrain from consuming alcohol or caffeine throughout the two study weeks). Exclusion criteria were diagnosed sleep disorders, prevailing sleep problems (Epworth Sleepiness Scale [ESS] score > 10; Pittsburgh Sleep Quality Index [PSQI] > 5,^31,32^) excessive smoking or caffeine consumption, the use of psychoactive medication, a history of or current substance use disorder (excluding tobacco), and extreme chronotypes (Morningness–Eveningness Questionnaire [D-MEQ] scores < 42 and > 58;^33,34^). In total, eight men (49.3 ± 3.8 years) with 18.9 ± 7.2 years of professional truck driving experience participated in the study. Screening revealed no excessive daytime sleepiness (ESS: 8.6 ± 2.0), no extreme chronotype (D-MEQ: 57.8 ± 4.6) and good subjective sleep quality (PSQI: 4.1 ± 0.6) in these participants.

The study strictly followed all relevant legal obligations and the principles of the 1964 Declaration of Helsinki. The study was approved by the ethical review committee of the University of Regensburg (Regensburg, Germany; reference number, 16-101-0327). All participants provided written informed consent before study inclusion.

### Technical specifications of DS

**Figure 2:**
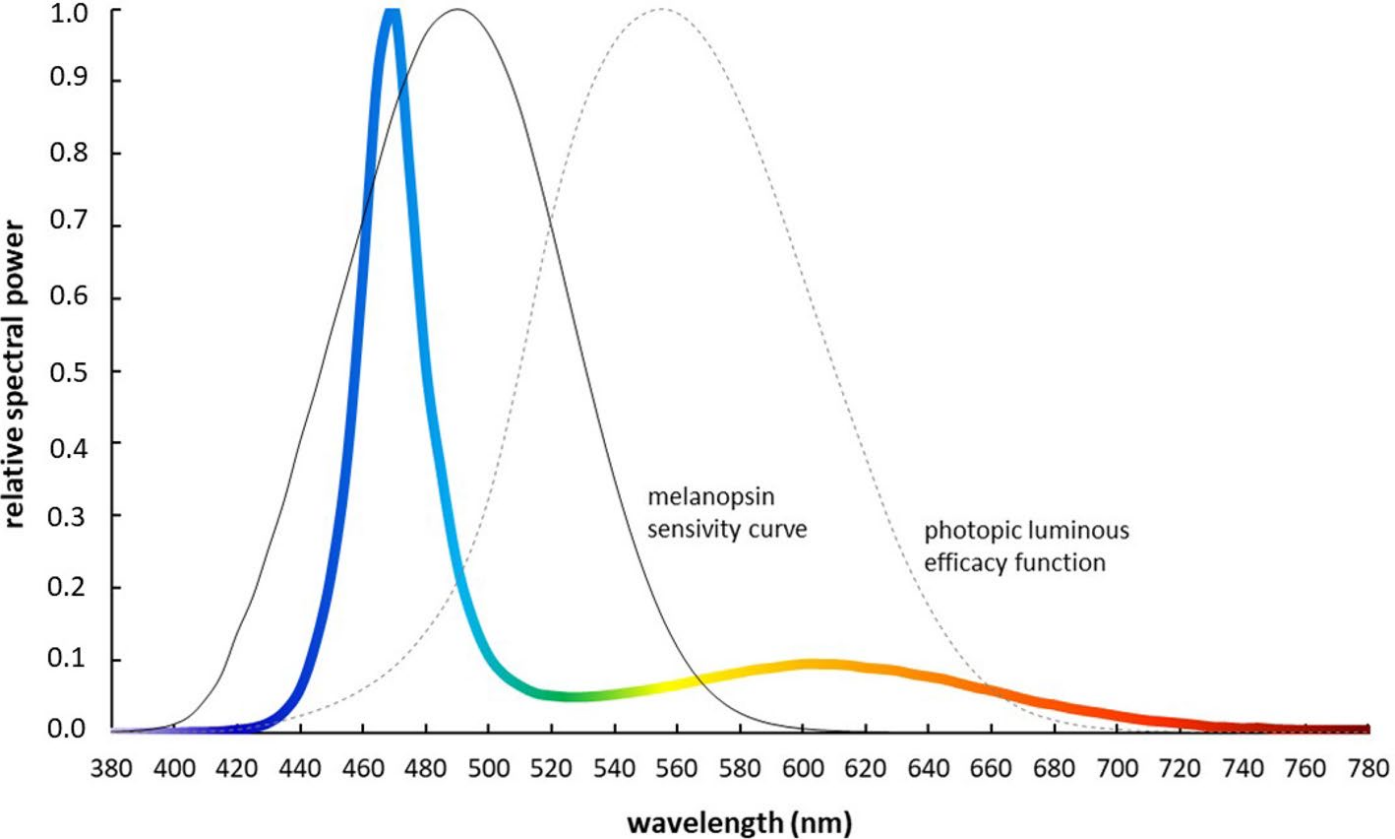
Normalized emission spectrum of the DS lighting system.

To avoid negative visual side effects while driving, additional in-cabin illumination was carefully specified before beginning the study. For this, the acceptance of different lighting scenarios in the driver’s cabin while driving at different illuminance levels and light spectra was investigated in an earlier, unpublished pilot study with another eight truck drivers (Supplementary Materials D). Based on these results, a luminaire was developed that emitted diffuse blueish-white light with high melanopic efficacy (3.64; see International Commission on Illumination [CIE] S 026/E:2018^35^) using 44 blue LEDs (peak wavelength, 472 nm; full width at half maximum, 25 nm) and 88 warm-white LEDs (2766 Kelvin color temperature; color rendering index = 82; CIE color space x/y coordinates = 0.456/0.412). The spectral light output of the DS was not changed throughout the study. Figure 2 shows the normalized emission spectrum of the DS and two sensitivity curves of human eyes related to visual and non-visual light effects (spectral data are given numerically in Supplementary Materials A.2, Table A.1).

When DS was switched on with full power, the light emitting surface reached a mean luminance of 22.680 cd/m^2^. Moreover, the mean luminance of highly reflective white material installed vertically nearby the luminaire was 1200 cd/m^2^. These high-luminance in-cabin areas were located outside the field of view of participants when driving the truck.

When the study participants reclined their chairs for light exposure during breaks in the morning and afternoon (DS^++^), illuminance at eye level reached a mean of 550 lx as measured during the study test phases. During driving with DS illumination (DS^+^), the maximum illuminance at eye level reached 400 lx during real driving conditions (under laboratory conditions, the calibrated luxmeter was positioned vertically at a distance of 87 cm from the ceiling, simulating a driver height of 187 cm; in this setting, the maximum illuminance was 377 lx). Figure 1a illustrates how the additional in-cabin illumination system applied light from above to reach the eyes of the truck drivers.

Light emission in DS^+^ was automatically and continuously dimmed during driving based on the illuminances measured at the windshield, measured with a photosensor (Adafruit Lux sensor TSL2591; Adafruit Industries, LLC, New York, USA). In addition, a LuxBlick 2.0 photosensor was placed at the same position to allow for a direct comparison between the illuminance at eye level (measured by a second LuxBlick 2.0 photosensor) and the outside illuminance (Fig. 1b). When the measured windshield illuminance reached 50 lx, the DS was switched on and additionally generated 40 lx blueish white light at eye level. The light output of the DS during driving was gradually increased (according to the lighting control curve; Supplementary Materials A.1, Fig. A.2) to the maximum level of the DS^+^ condition (400 lx) at a windshield illuminance of 1200 lx. The DS system was kept at maximal light output when windshield illuminance levels exceeded 1200 lx. If the windshield illuminance fell below 50 lx, the DS was not switched off immediately, but the light output was further dimmed linearly to 0 lx at a windshield illuminance of 20 lx. At lower windshield illuminances, the DS was switched off. This hysteresis effect in the light control strategy of DS^+^ (i.e., switching on at 50 lx and switching off at 20 lx windshield illuminance) aimed to reduce the amount of switching on maneuvers of the illumination system and thus should reduce distractions to the truck drivers while driving under DS^+^. To control the LEDs contained in the DS, a Raspberry Pi and a digital multiplex controller (Chromoflex Pro CV; Barthelme GmbH, Nuremberg, Germany; Open DMX Ethernet; ENTTEC Ltd., London, UK) were utilized.

As dimming levels in DS^+^ were recorded continuously, melanopic equivalent daylight illuminances (MEDIs)^35^ could be estimated by applying the following two assumptions: (i) daylight within the truck is similar to the CIE standard illuminant D65 (CIE S001:1986^36^) and (ii) only 14% of illuminance measured at the windshield reaches the participants’ eyes, as measured during the study. Consequently, MEDI at the driver’s eyes was estimated by adding the MEDI generated by the DS and the MEDI caused by in-cabin daylight reaching the eyes of the participants.

### Statistics

The study was conducted as an experimental single-blind field study with a three-factorial (Intervention, Measurement Time, Weekday) repeated-measures design using an active placebo control condition. Due to the fact that the factor Weekday was not significant, two-factorial repeated-measures ANOVAs with the factors Intervention and Measurement Time were run for KSS, PVT reaction speed, and the number of lapses on the PVT. For these analyses, data recorded on all workdays except Thursday were subjected to analyses. Before running the ANOVA, data were checked for outliers, normality, and sphericity; in cases of sphericity violations, a Greenhouse–Geisser correction of the significance level was applied. All post-hoc pairwise comparisons were run with Bonferroni-corrected significance levels. In addition to the ANOVA models, we also considered non-parametric Bonferroni-corrected paired-samples statistical tests to justify their use. Both analysis procedures yielded the same significant study results. Therefore, only the results of the ANOVAs are presented.

Acceptance data were subjected to non-parametric Friedman tests. Whenever test results were significant, pairwise Wilcoxon tests with Bonferroni-corrected significance levels were calculated. For descriptive analyses, mean, standard deviation, standard error, median, and interquartile range were calculated. All graphs show means and standard errors unless otherwise specified.

Intervention effects on subjective sleep quality parameters were determined by applying t-tests for two dependent samples. Outliers were identified by searching for data beyond three standard deviations below and above the mean. No outliers were found using this procedure. All analyses were performed in SPSS software (v28.0; IBM Corp., Armonk, NY, USA) with a significance level of 5% and two-sided testing.

Interventions using bright light during the day have shown small to medium effect sizes on simple alerting tasks^37^. Therefore, an a priori power analysis was performed using G*Power version 3.1.9.7 to determine the minimum sample size required for the present study^38^. To achieve a power of 80% for the detection of a small to medium effect (effect size = 0.04) at a significance level of α = 0.05, an assumed correlation of 0.80 between repeated measures, and a non-sphericity correction factor of 1 for a two-factorial ANOVA with repeated measures, eight participants were required.

### Informed consent

Written informed consent was obtained from the participants for the publication of identifying images in an online, open-access journal.

## Results

### Feasibility of DS for supplementing in-cabin daylight

#### Daytime light exposure

In-cabin illuminances were measured continuously from 08:30 to 15:45 close to the eyes of the truck drivers with a wearable device (LuxBlick 2.0^39^). To better account for non-visual effects of light on the melanopsin photoreceptor system, the MEDI (CIE S 026/E:2018^35^) at eye level was additionally estimated.

#### *Light exposure during driving rest periods* (DS^++^)

DS^++^ was applied before and after test drives (from 09:00 to 10:00 and from 14:00 to 15:40). In the morning, participants were exposed to 531 ± 221 lx under DS^++^ compared to 47 ± 60 lx in the placebo condition. This corresponded to an estimated mean 1459 MEDI under DS^++^ versus 19 MEDI in the placebo intervention. Similarly, participants were exposed to higher illuminances in the afternoon under DS^++^ compared to the placebo (DS^++^: 538 ± 253 lx, estimated mean 1478 MEDI; placebo: 28 ± 55 lx, estimated mean 11 MEDI).

#### *Light exposure during driving (DS*^+^*)*

**Figure 3:**
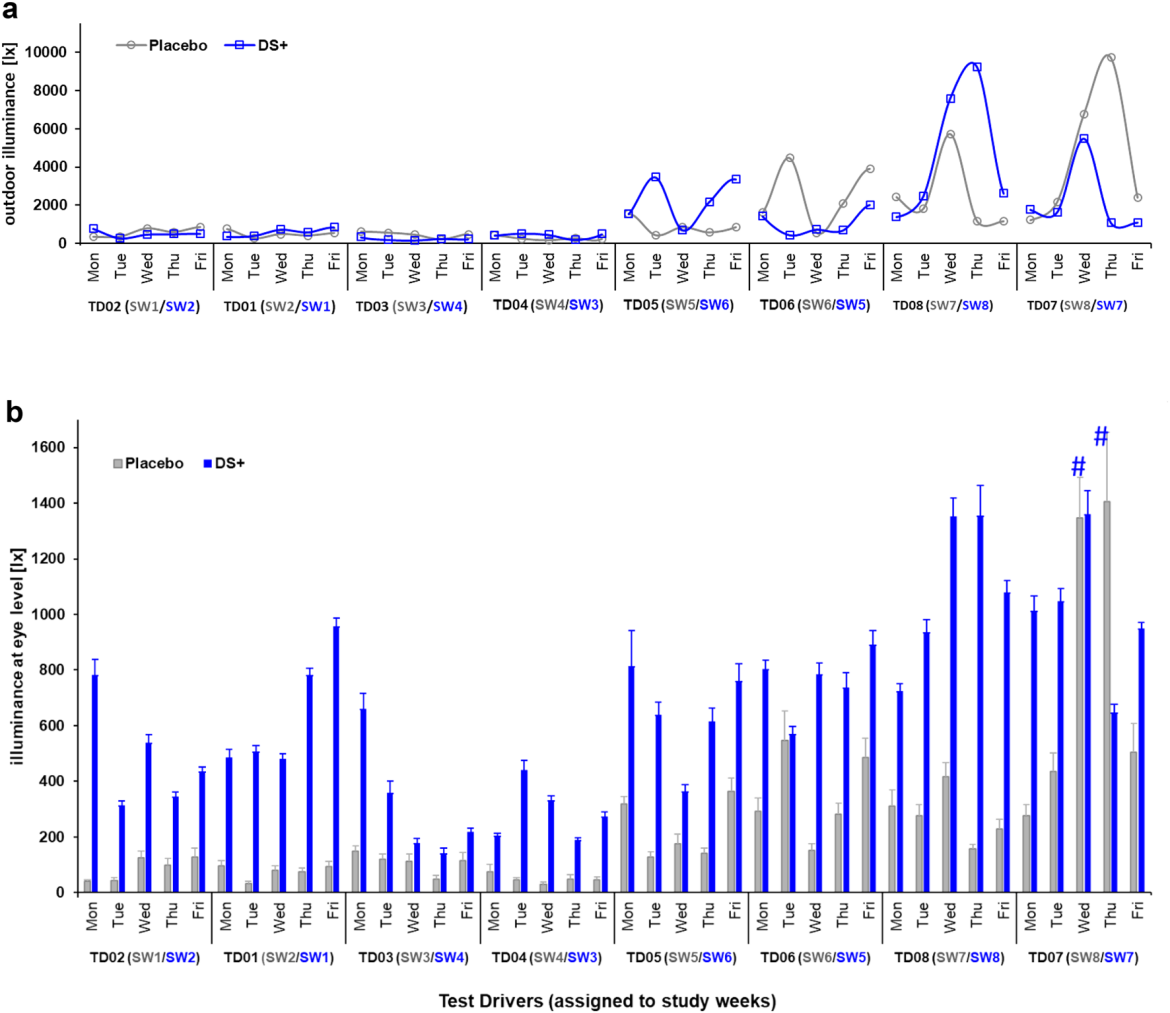
Light conditions during driving; (**a**) mean outdoor illuminance (estimated with a photosensor behind the windshield) is depicted during the driving periods for the placebo and the active light (DS+) condition over the course of eight study weeks (SW) from the end of November until early February; (**b**) mean illuminances at eye level are compared for each corresponding study weekday of the placebo and the DS+ condition; error bars indicate standard errors of the means; on two bright days (marked with hashtags), illuminances at eye level were above 1,000 lx in the placebo condition.

During the 4-h driving periods, truck drivers were exposed to 650 ± 379 lx under DS^+^ compared to 247 ± 374 lx in the placebo condition with no additional artificial in-cabin light, resulting in a 2.5-fold increase in photopic stimulation (Fig. 3). Moreover, the drivers were exposed to an estimated average of 1,346 MEDI under DS^+^ compared to 247 MEDI in the placebo condition, indicating an increase in melanopic stimulation by a factor of 5.4. Without additional in-cabin lighting (i.e., in the placebo condition), only 14% of the windshield illuminances reached the drivers’ eyes.

**Figure 4:**
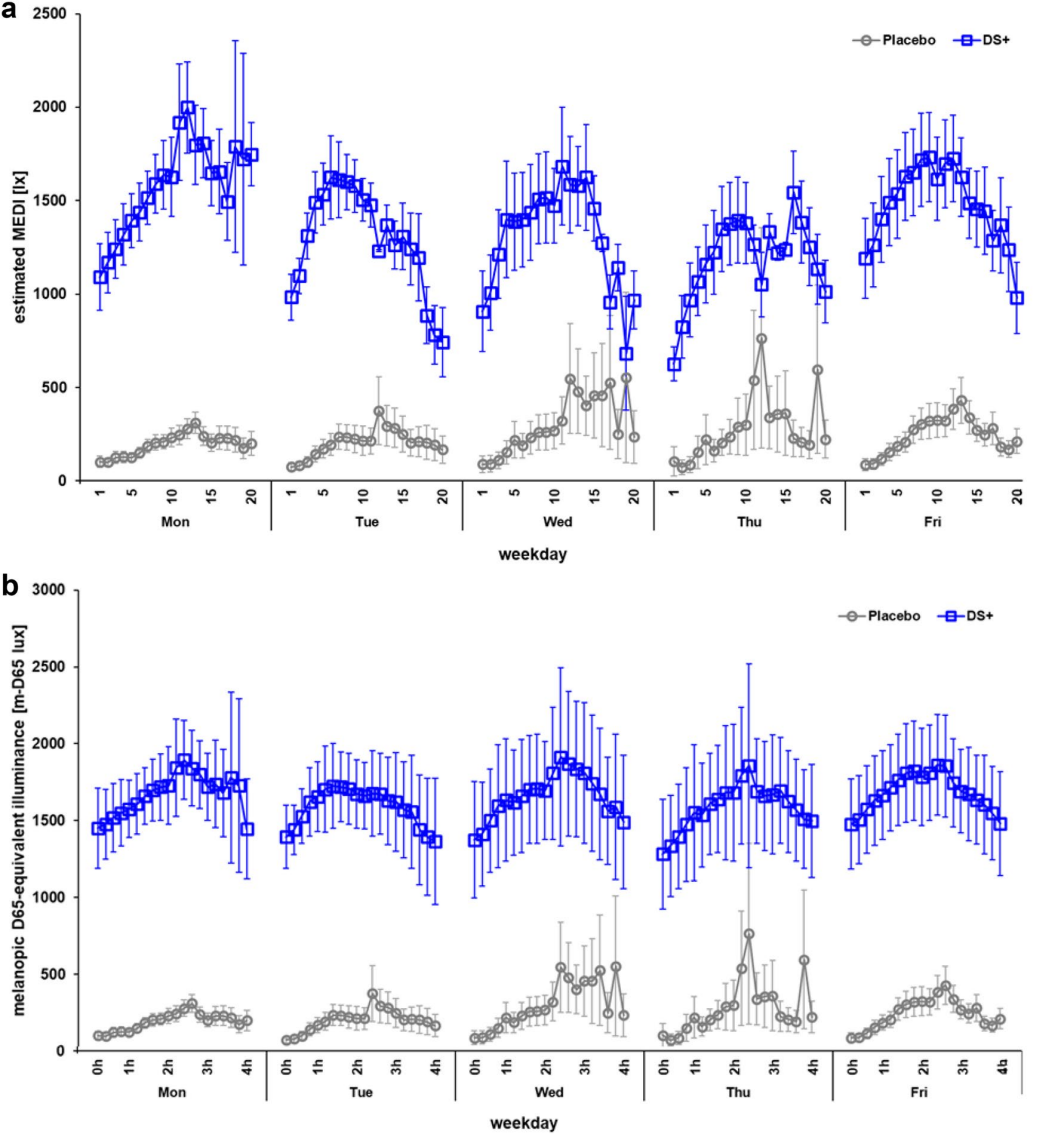
Light exposure during the 4-h test drives; (**a**) mean illuminances at eye level; (**b**) estimated mean melanopic daylight equivalent illuminances (MEDIs) at eye level; data are shown per hour (divided by 5 separate 12-min intervals) across five weekdays; the error bars show the standard errors of the mean.

In the second half of the eight study weeks starting in early January, outdoor illuminances varied considerably, barring two extremely bright days (during the placebo condition) in the last study week in early February (Fig. 3; study week 8). Figure 4 shows all measured illuminances at eye level and estimated MEDIs with high temporal resolution (12-min intervals) across the 4-h test drives.

### Comfort ratings for the interventions

At the end of the study week, the truck drivers provided comfort ratings for each intervention and were asked to share any negative side effects. Table 2 summarizes the comfort ratings for the different intervention components while driving, including DS and the two co-occurring placebo components, inactive UV light exposure and the mock air-refreshing system.

**Table 2:** Comparison of comfort ratings (medians and interquartile range) between the active light condition DS^+^ and the placebo intervention, which consisted of two components (inactive UV light exposure and placebo air freshening system).

COMFORT RATINGS	DS^+^ *Mdn [IQR]*	UV *Mdn [IQR]*	AIR *Mdn [IQR]*	Friedman ANOVA	*Post-hoc comparisons*
**Semantic Differential** (7-point Likert scale: 1 = *very*…; 7 = *very*…; 4 = *neither nor*)					
*pleasant–unpleasant*	1.0 [1.0–2.0]	4.0 [4.0–4.0]	2.5 [1.8–4.0]	.002	.027*/ns°
*sleep-inducing–activating*	6.5 [6.0–7.0]	4.0 [4.0–4.0]	4.0 [2.0–4.0]	.004	.030*/ns°
*familiar–unfamiliar*	7.0 [4.0–7.0]	4.0 [4.0–4.0]	4.0 [4.0–4.5]	ns	n.a
*unobtrusive–obtrusive*	3.5 [1.8–4.0]	4.0 [1.0–4.0]	3.0 [1.0–4.0]	ns	n.a
**Rating for interventions** (5-point Likert scale: 1 = *not at all*; 5 = *very*)					
*…improved my well-being while driving*	4.5 [4.0–5.0]	1.5 [1.0–3.0]	2.0 [1.0–3.0]	.001	.033*/.033°
*…improved my fitness*	4.0 [3.8–5.0]	1.5 [1.0–3.0]	1.0 [1.0–3.0]	.002	ns*/.033°
*…disturbed me while driving*	1.0 [1.0–1.0]	1.0 [1.0–1.0]	1.0 [1.0–1.0]	ns	n.a
**Overall rating for interventions** (6-point Likert scale: 1 = *not at all*; 6 = *absolutely*)					
Recommendation to other truck drivers	5.5 [5.0–6.0]	3.0 [1.8–3.3]	3.0 [2.8–3.0]	.001	.033*/ .030°
**Evaluation of the interventions** (6-point Likert scale: 1 = *very good*; 6 = *insufficient*)					
Grades (i.e., school grading scale)	1.3 [1.0–1.6]	3.3 [3.0–4.1]	3.0 [2.8–3.4]	.002	.036*/ns°

*Bonferroni-corrected post-hoc comparisons (Wilcoxon test) between DS and UV; ° Bonferroni-corrected post-hoc comparisons (Wilcoxon test) between DS and AIR; all Bonferroni-corrected post-hoc comparisons between UV and AIR did not reach significance; AIR, placebo air refreshment system; DS^+^, active light system; UV, inactive ultraviolet light exposure system; n.a., not applicable; IQR, interquartile range; Mdn, Median.

In contrast to the placebo components, the truck drivers rated DS^+^ as more “pleasant” and “activating” on a semantic differential (list of bipolar adjective pairs; range from 1 to 7; Table 2). On a 5-point Likert scale, DS^+^ was perceived as significantly more effective in improving well-being than the other intervention components. In addition, DS^+^ improved the truck drivers’ subjective self-assessment of their fitness. Furthermore, participants did not perceive DS^+^ as intrusive and did not rate the adjective pairs “familiar–unfamiliar” and “unobtrusive–obtrusive” differently for the three intervention components (Table 2).

Finally, truck drivers rated DS^+^ with an average score of 1.3 (graded from 1 = *very good* to 6 = *insufficient*), and thus significantly better than the placebo intervention components (UV, 3.8; AIR, 3.1). With an average recommendation score of 5.5 (on a scale of 1 = *not at all* to 6 = *absolutely*), the participants strongly recommended the DS^+^ system to other truck drivers.

Importantly, no study participants reported any negative visual side effects (e.g., restrictions due to glare, dazzling, or eye irritation) while driving in the DS+ or placebo conditions. The full distribution of the ratings results is provided in the Supplementary Materials C.1, Table C.1.

### Efficacy of DS on daytime alertness before and after driving

On four of the five study days in the DS^++^ condition (except for Thursday morning), truck drivers were exposed to bright light before the drives started and after they ended, and subjective and objective alertness data were recorded.

#### Effects on subjective alertness

**Figure 5:**
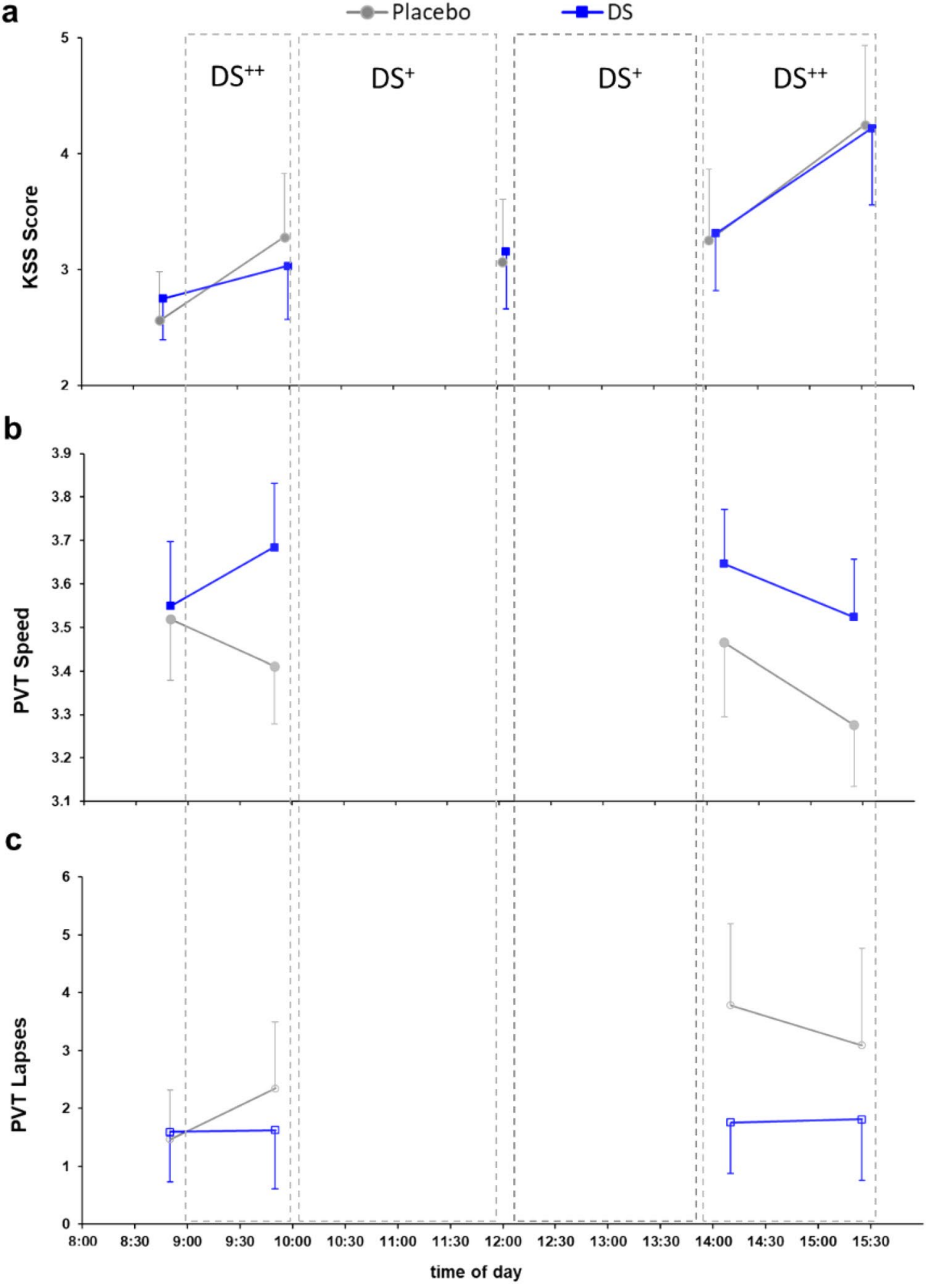
Subjective measure of sleepiness and objective measures of alertness during driving rests in the morning and afternoon; (**a**) self-reported sleepiness assessed with the Karolinska Sleepiness Scale (KSS); (**b**) reaction speed (in s^−1^) on the Psychomotor Vigilance Task (PVT); (**c**) number of lapses on the PVT; error bars indicate 95% confidence intervals of the mean.

The subjective sleepiness measures (KSS) are shown in Fig. 5a. We observed a significant interaction for morning KSS scores (F [1,31]  = 4.534, *p* = 0.041, partial η^2^ = 0.128). Post-hoc tests showed that the KSS values did not differ between the two interventions at both morning measurement times (both *p* > 0.05). However, subjective sleepiness increased over time in the placebo condition (08:45, 2.56 ± 0.21; 09:55, 3.28 ± 0.27; *p* < 0.001), while it remained at the same level in the DS^++^ condition (08:45, 2.75 ± 0.17; 09:55, 3.03 ± 0.23; *p* = 0.372).

A second ANOVA was run to determine the effects of DS^+^ on subjective sleepiness during the test drives by analyzing data from three measurement points (just before the drive started [09:55], during the drive break [12:00], and immediately after the drive ended [14:05]). We did not observe significant interactions or main effects of Intervention or Measurement Time on KSS scores (all *p* > 0.05).

A third ANOVA examined the effects of DS^++^ on subjective sleepiness in the afternoon after driving ended. We did not find a significant interaction effect or a main effect of Intervention on KSS scores (both *p* > 0.10). However, subjective sleepiness significantly increased over time in the afternoon under both study arms (14:05, 3.38 ± 0.28; 15:35, 4.23 ± 0.33; F [1, 31]  = 18.140, *p* < 0.001, partial η^2^ = 0.369). Thus, the highest sleepiness ratings (scores ranged between *rather alert* and *neither alert nor sleepy*) were obtained at the end of the simulated working day in both study conditions.

#### Effects on objective measures of alertness

A PVT was performed twice in the morning before driving and twice in the afternoon after driving. In the morning, the PVT took place just before DS^++^ started (at 08:45) and after 45 minutes of exposure to DS^++^ (at 09:45). In the afternoon, the PVT was performed immediately after the start of the exposure to DS^++^ (at 14:05) and after 90 minutes of DS^++^ exposure (at 15:15). Due to skewed data, the reciprocal reaction time (1/RT; i.e., PVT reaction speed) was subjected to statistical analysis.

#### PVT reaction speed

PVT reaction speeds are shown in Fig. 5b. In the mornings, we observed an increase in reaction speeds under DS^++^ and a decrease in the placebo condition between the two tests. A two-way ANOVA revealed a significant interaction effect during the morning under DS^++^ (F [1, 31]  = 25.324, *p* < .001, partial η^2^ = .450). Both interventions did not differ at 08:45 (DS^++^ 3.55 ± 0.07 s^−1^; placebo: 3.52 ± 0.07 s^−1^; *p* = .999) but showed a significant difference at 09:45 (*p* < .001) with higher reaction speed under DS^++^ (3.68 ± 0.07 s^−1^) compared to the placebo condition (3.41 ± 0.06 s^−1^). Moreover, we observed a significant increase in reaction speed under DS^++^ and a significant decrease in reaction speed under the placebo condition (both *p* < .05).

In the afternoon, there was a comparable decline in reaction speed in both conditions, but on different levels. There was no significant interaction effect of DS^++^ (F [1, 31]  = 1.509, *p* < .229, partial η^2^ = .046). However, there were significant main effects of Intervention (F [1, 31] = 20.446, *p* < .001, partial η^2^ = .397) and Measurement Time (F [1, 31] = 26.839, p < .001, partial η^2^ = .464), with higher reaction speed under DS^++^ (3.58 ± 0.06 s^−1^) than under the placebo condition (3.37 ± 0.07 s^−1^) and higher reaction speed at the first measurement time at 14:05 (3.55 ± 0.07 s^−1^) than at the second measurement time at 15:15 (3.40 ± 0.06 s^−1^).

#### Number of lapses on PVT

The number of lapses on the PVT across the four measurement points during the day are depicted in Fig. 5c. A two-way ANOVA showed a significant interaction effect of DS^++^ in the morning (F [1, 31] = 6.293, *p* = .018, partial η^2^ = .169). Both interventions did not differ at the first measurement point at 08:45 (*p* = .999) but showed a significant difference at 09.45 (*p* = .030), with fewer lapses under DS^++^ (1.63 ± 0.50) than the placebo condition (2.34 ± 0.56). Moreover, there was a significant increase in the number of lapses in the placebo condition between these measurement times (*p* = .005). In addition, we observed no significant interaction or main effects of Measurement Time on the number of lapses under DS^++^ in the afternoon (Interaction: F [1, 31] = 0.459, *p* = .503, partial η^2^ = .015; Measurement Time: F [1, 31] = 0.413, *p* = .525, partial η^2^ = .013). However, the main effect of Intervention reached significance (F [1, 31] = 7.952, *p* = .008, partial η^2^ = .204), with fewer lapses under DS^++^ (1.78 ± 0.47) than under the placebo condition (3.45 ± 1.00).

### Carry-over effects of DS beyond the working day

To assess any potentially adverse activating effects of DS in the evenings, subjective alertness measures and melatonin levels were assessed on three study days (Monday, Wednesday, and Friday) on an hourly basis from 17:00 to 22:00 as well as once immediately before going to bed, at 22:25. Results from two-factor repeated-measures ANOVAs with the factors Intervention and Measurement Time are reported here together with results of sleep quality assessments.

#### Effects on evening alertness levels

**Figure 6:**
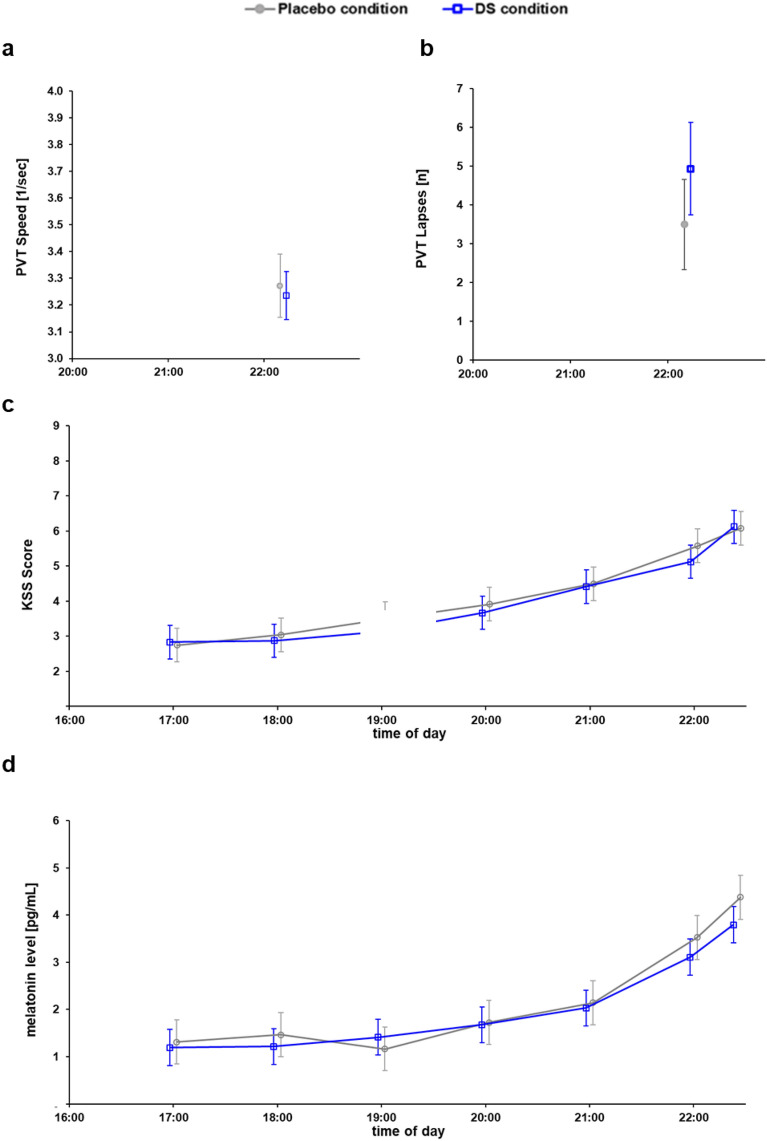
Carry-over effects of DS beyond the working day; (**a**,**b**) two objective alertness measures of the Psychomotor Vigilance Task (PVT) at 22:00. (PVT reaction speed and number of lapses); (**c**) self-reported sleepiness using the Karolinska Sleepiness Scale (KSS); (**d**) saliva melatonin levels (pg/mL) in the evening.

Figure 6c shows similar significant increases in self-reported sleepiness levels (KSS ratings) in both study conditions over time (F[6,138] = 45.874, *p* < 0.001, partial *η*^*2*^ = 0.666), starting with *alert* (KSS score: 3) to *some signs of sleepiness* (KSS score: 6) before bedtime (DS: from 2.75 ± 0.24 to 6.08 ± 0.45; placebo: from 2.83 ± 0.20 to 6.13 ± 0.34). No significant differences of Intervention or interaction effects were found between both experimental conditions (Intervention: F[1,23] = 0.596, *p* = 0.448; Interaction: F[6,138]  = 0.710*, p* = 0.642).

In addition, objective alertness before bedtime was measured with the PVT on Monday and Friday at 22:00. By applying a t-test for two dependent samples, we observed no differences in reaction speed between the two interventions (t[15] = 0.107, *p* = 0.748; DS: 3.23 ± 0.09; placebo: 3.27 ± 0.12). In contrast, the number of lapses on the PVT was different (t[15] = 5.064, *p* = 0.040) with more lapses under DS (4.94 ± 1.20) than under the placebo condition (3.50 ± 1.17) (Fig. 6a,b).

#### Effects on evening melatonin levels

Saliva melatonin was sampled on three evenings (Monday, Wednesday, and Friday). As shown in Fig. 6d, mean saliva melatonin levels increased from 17:00 to 22:25 in both study conditions (DS: 1.19 ± 0.18 pg/mL to 3.80 ± 0.44 pg/mL; Placebo: 1.31 ± 0.13 pg/mL to 4.38 ± 0.53 pg/mL; F[6,138] = 37.341, *p* < 0.001, partial η^2^ = 0.619), with no significant effect of Intervention or Interaction (Intervention: F[1,23] = 3.518, *p* = 0.073; Interaction: F[6,138] = 1.950, *p* = 0.077).

#### Effects on sleep quality

Truck drivers were asked to document their sleep onset and offset times and rate their subjective sleep quality after each of the five study nights (Monday to Friday) in both study conditions.

Subjective sleep duration did not differ between the two interventions (DS: 389 ± 53 min; placebo: 389 ± 57 min; *p* = 0.998). In addition, overall subjective sleep quality as measured by the SSA did not differ between conditions (DS: 30.5 ± 9.2, placebo: 31.0 ± 6.2; *p* = 0.348). This remained true for the three SSA subscales of awakening quality (DS: 13.0 ± 4.3, placebo 12.9 ± 3.5; *p* = 0.880), somatic complaints after awakening (DS: 5.7 ± 1.2, placebo: 5.7 ± 0.9; *p* = 0.781), and specific sleep quality (DS: 11.9 ± 3.9, placebo: 12.6 ± 3.0; *p* = 0.348).

Results of the objective sleep quality measurements using PSG twice per study week (Monday and Friday) are shown in the Supplementary Materials (C.3 and Table C.3). In general, the data quality was limited, as the PSG data were often either missing or noisy (e.g., electrodes lost contact during the night). Based on the existing results, however, DS showed no relevant negative effects on global sleep parameters.

## Discussion

The present field study evaluated the feasibility and efficacy of a daylight-supplementing in-cabin lighting system to improve daytime alertness in truck drivers, utilizing objective measures of alertness. The study was performed in a naturalistic setting mimicking real working and living conditions of truck drivers in a wintry Arctic environment with low daylight levels. In the present study, the truck served not only as a workplace but also as a living space.

The main findings indicated that exposure to supplemented light through DS significantly increased alertness without any adverse effects during driving. Objective alertness, as measured using the PVT, notably improved with bright light exposure before and after driving. Furthermore, participants reported improved subjective alertness in the morning. There were no observed negative effects on evening levels of melatonin, sleepiness, or nighttime sleep quality, suggesting that DS did not adversely affect sleep patterns.

Currently, there is insufficient knowledge about transport users’ light exposure while driving or sitting in a vehicle. Two recently published studies measuring light exposure using wearable devices provided initial evidence that passengers^40^ and drivers^29^ are exposed to low light levels. In these studies, multiple factors influenced actual in-cabin light exposure such as the sitting and viewing position of the passenger, vehicle type (e.g., car, bus), environmental obstructions, travel direction, time of day, season, and geographic location.

Although truck cabins consist of extensive windows with high light transmission^41^, the results of the present study supported the assumption that only a fraction of the light (i.e., 14% of the illuminance measured behind the windshield) actually reaches truck drivers’ eyes. This sums to an average illuminance of 247 lx at eye level while driving, which is comparable to light exposure levels in stationary indoor (working) spaces^42–44^. Particularly in seasons with little daylight, professional drivers are thus exposed to low diurnal light levels at their workplaces. On bright, sunny summer days (with outdoor light levels of more than 10,000 lx for several hours), supplementary in-cabin light exposure may not be necessary to enhance drivers’ alertness levels, but in winter months, with shortened daylight periods and lower light levels, additional in-cabin light may be beneficial.

Due to its small form factor and controllability, LED technology is widely used in interior car lighting^45^. In-cabin lighting has been primarily used in passenger cars and fulfills visual and aesthetic needs, but it is not often designed to combat safety issues. In-cabin lighting for trucks, especially when used to increase light exposure to truck drivers work and to generate acute alerting effects while driving or during breaks, has not yet been investigated. While driving under low daylight conditions, the DS system used here significantly increased drivers’ daytime light exposure while driving (increased photopic illumination by a factor of 2.6) as well as before and after driving. These numbers are based on a photopic evaluation of light stimuli and do not consider non-visual light effects due to the spectral sensitivity of melanopsin-containing intrinsically photosensitive retinal ganglion cells. As the melanopic efficacy of DS was almost three times higher than that of natural daylight, retinal ganglion cells received even stronger stimulation when using the system than under the low in-cabin light levels in the Arctic winter months (increased by a factor of 5.4).

Importantly, test drivers in the present study perceived the DS to be comfortable and did not report any negative side effects while driving. We further could not observe negative carry-over effects of DS on melatonin levels in the evening, detrimental (i.e., arousing) effects on subjective and objective measures of alertness right before bedtime, or effects on nighttime subjective sleep quality parameters. These results supported the notion that this daylight-supplementing in-cabin lighting system is feasible for use in trucks when driving in low daylight levels. Recently published systematic reviews of bright light exposure provide good evidence for enhanced subjective alertness acutely triggered by daytime bright white light exposure^24^ but have reported only small effects of bright light on objective alertness measures^25^. In the present study, we found acutely improved objective measures of alertness after blueish white light exposure in the morning and afternoon, accompanied by enhanced subjective alertness in the morning.

In the present study, subjective sleepiness levels increased gradually throughout the day in both conditions, reaching their highest point after the PVT during the late afternoon rest period. Notably, in the DS^++^ condition, where the PVT was conducted before the morning test drive, sleepiness scores remained stable while reaction speed improved. However, in the placebo condition, both sleepiness and reaction speed worsened. Although the KSS-sleepiness scores increased similarly during the late afternoon rest period, there were significant differences between interventions in terms of PVT reaction speed (higher in DS^++^) and number of lapses (lower in DS^++^). These results are consistent with Kaida et al.'s (2006) study^46^, which found moderate correlations between subjective sleepiness measures (KSS) and mean PVT reaction time (*r* = .57) and number of lapses in PVT (*r* = .56).

Subjective measures of sleepiness did not deteriorate when alertness tests were conducted in bright light and with low sleepiness levels (in the morning). In contrast, objective measures of alertness appear to benefit from exposure to bright light, particularly in reducing the number of lapses in the PVT. This effect was moderated to a lesser extent by the level of subjective sleepiness. Further studies are needed to clarify the complex relationship between objective measures of alertness, subjective measures of sleepiness, time of day, and cumulative workload.

Due to technical shortcomings, we were not able to measure direct alerting effects of the DS on physiological arousal (alpha spindle rate) and driving performance. To the best of our knowledge, two studies have reported beneficial diurnal effects on these parameters from additional in-vehicle lighting in passenger cars. In one study, which also utilized the DS system used here, alpha spindle rates acutely decreased during real driving on a motorway in the morning^29^. In a second driving simulator study with chronically sleep-restricted drivers^47^, combining bluish-green light exposure (500 nm; 506 lx at eye level) and caffeine administration (100 mg caffeinated gum) improved driving performance (i.e., lateral lane position), neurophysiological alertness (EEG alpha power), and subjective sleepiness. Thus, in-cabin lighting, at least in the morning, seems to be a promising intervention to increase physiological alertness and improve driving performance. Future studies are needed to demonstrate whether these effects also occur in professional truck drivers.

## Limitations

The present study comes with some limitations. First, data were recorded in a naturalistic environment with varying external factors (e.g., weather conditions, traffic volume), potentially masking effects of the intervention. Non-visual light effects have mostly been investigated in highly controlled laboratory conditions with low ecological validity, and this study provided novelty in investigating diurnal alerting effects of the DS under naturalistic working conditions. Second, this study was conducted in the winter in low daylight levels, and the effects of DS on under bright outdoor light conditions remain unknown. Third, the short data collection period limited the included data to eight male professional truck drivers. Although an a priori power analysis revealed that the inclusion of eight participants was sufficient to detect small to moderate effects, these results are preliminary and should be interpreted with caution. Further studies with larger samples are needed to corroborate our results. Due to gender bias in recruitment, the results cannot easily be generalized to the general population. Fourth, the quality of the recorded neurophysiological data was often insufficient, as EEG as well as PSG data were occasionally either missing (lost electrode contact) or noisy (movement and eye blink artifacts). Moreover, driving performance data quality was also low because the wintry road conditions did not allow us to reliably determine lane keeping behavior or the lateral positions of the trucks. Consequently, we were not able to determine some effects of the DS while driving, and the reported alerting effects must primarily be assigned to the light interventions taking place before and after the drives.

## Conclusions

This field study demonstrates that DS increased daytime light exposure in low daylight conditions and might improve alertness in truck drivers before and after driving, such as during driving rest periods. Enhancing alertness in truck drivers by providing light exposure in their natural working environment could be a promising way to acutely manage drowsiness while working under monotonous conditions with low daylight levels (e.g. doing digital work or paperwork in the cabin). Moreover, bright light can be used during breaks as an alerting alternative to napping, caffeine consumption, or walking outside when these activities are not possible. The results of the present study provide a basis for further research investigating the acute effects of daylight supplementing in-cabin lighting in the automotive context to prevent driver drowsiness, promote alertness, and improve road safety.

### Supplementary Information

Supplementary Information.

## Data Availability

Study data are available from the corresponding author on reasonable request.
