# Supplementary material for: Multimodal in-vehicle lighting system increases daytime light exposure and alertness in truck drivers under Arctic winter conditions
Source: Sci Rep. 2024 Apr 30;14:9925. doi: 10.1038/s41598-024-60308-y (PMC11061141; doi:10.1038/s41598-024-60308-y)
Supplement: Supplementary file 1 — Supplementary Information. [file 41598_2024_60308_MOESM1_ESM.pdf]

Multimodal in-vehicle lighting system increases daytime light exposure and alertness in truck drivers under Arctic winter conditions

Roland F.J. Popp* [1], Julia Ottersbach [1,2], Thomas C. Wetter [1], Sebastian Schüler [3], Siegfried Rothe [3], Daniel Betz [3], Siegmund Staggl [4], Markus Canazei [4]

[1] Department of Psychiatry and Psychotherapy, Center of Sleep Medicine, University of Regensburg, 93053 Regensburg, Germany

[2] Institute of Experimental Psychology, University of Regensburg, 93053 Regensburg, Germany

[3] Mercedes-Benz AG, 71059 Sindelfingen, Germany

[4] Department of Psychology, University of Innsbruck, 6020 Innsbruck, Austria

*Corresponding author: roland.popp@medbo.de

Appendix A: Technical details of the in-vehicle daylight-supplementing lighting system (DS) and the placebo condition

The multimodal in-vehicle lighting system "DS" (see Fig. A.1) was primarily developed to supplement light exposure in the truck while driving (DS^{+}). In addition, the system can be used as bright light application before and after driving (DS^{++}). The system is also capable of acting as a dawn simulator in the early morning before getting up (however, this was not a focus of the study and technical details are not reported here).

A.1 Light application and controlling DS while driving

By optimizing the technical aspects of DS in regard to geometry and reflectivity of the surface (e.g., by painting the beige storage door above the driver's head matte white), the maximal illuminance at eye level reached 400 lx during real driving with DS^{+} in the study condition (under laboratory conditions, illuminance levels reached 377 lx measured at a fixed height of 87 cm below the ceiling, directed vertically).

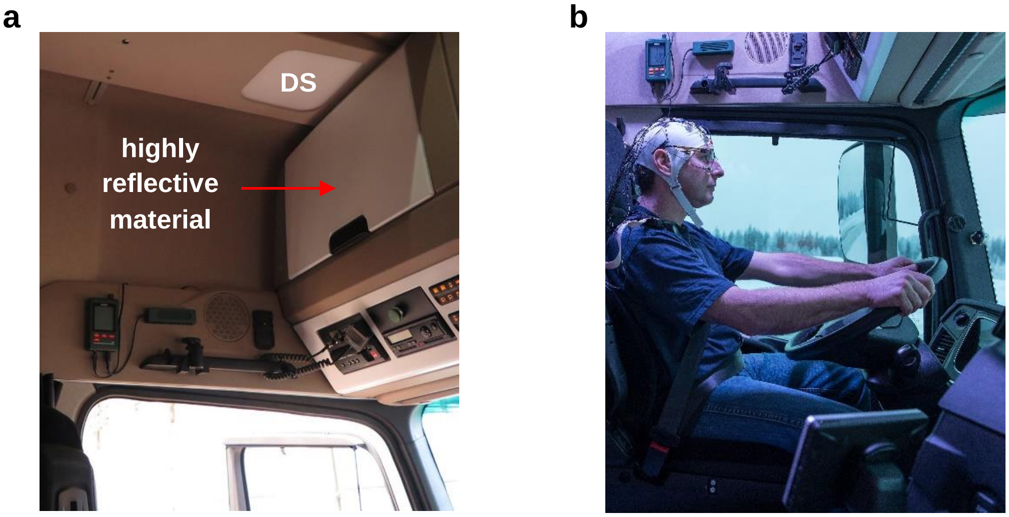

Figure A.1 The in-vehicle daylight-supplementing lighting system (DS); **a:** The light module of the DS lighting system (31 × 31 cm) and a highly reflective white material installed vertically nearby the DS to increase the amount of light reaching the eyes of the driver;
b: daylight supplementation while driving (DS^+).

In order to avoid dazzling or disturbing reflections on the windshield from the DS system during driving (i.e., in dim daylight conditions or when the truck is entering a tunnel), the inside illuminance was automatically adapted to the lighting conditions outside of the truck, which were measured using a high dynamic range digital photosensor attached vertically to the lower windshield (Adafruit Lux sensor TSL2591; Adafruit Industries, LLC, New York, USA; see Fig. 1 b in main text). The lighting adjustment was adapted every 50 ms (illuminance levels were based on the mean windshield illuminance during the previous 2.5 s using a sliding window) and controlled by a calibration curve (Fig. A.2).

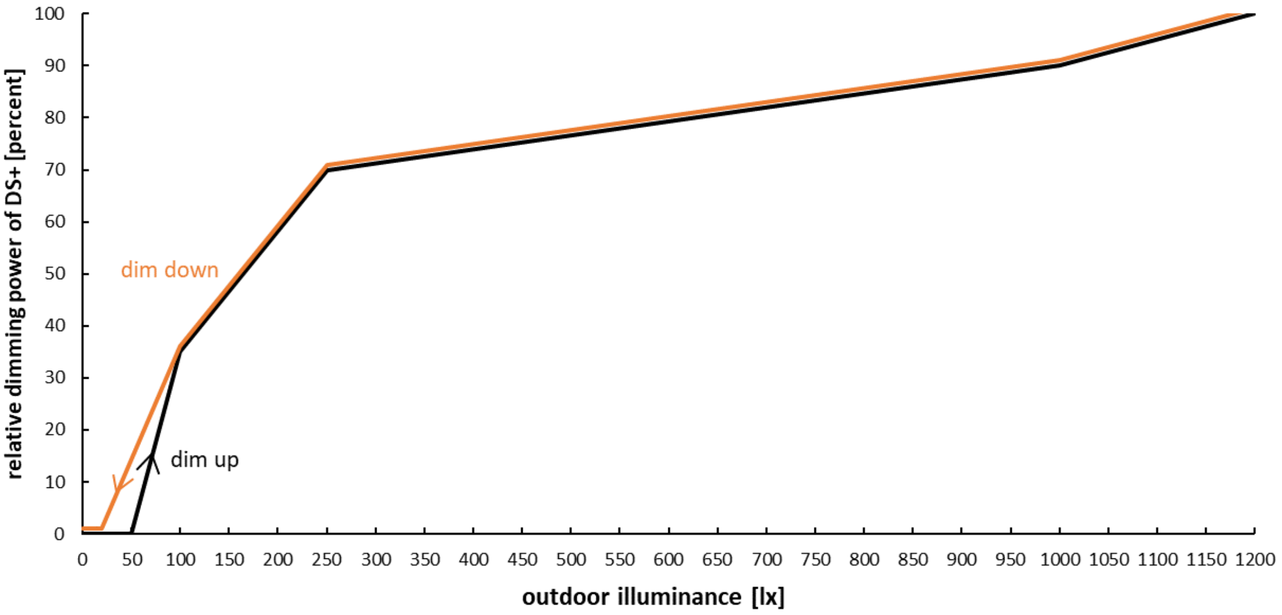

Figure A.2 The lighting control curve of DS; the curve shows the dimming percentage of DS^+ related to the outdoor illuminance level measured at the windshield of the truck; the dim down control strategy was chosen slightly different compared to the dim up strategy to increase visual comfort.

More technical details on the adaptive function of the DS can be found in a recent publication [1].

A.2 Technical specifications of DS

In addition to Figure 2 in the main manuscript, which demonstrates the normalized emission spectrum of the DS, Table A.1 summarizes numerical data on the spectral and photometric measures of the DS while driving (DS^+) or during the bright light application (DS^{++}) before or after driving. The reported measures follow recommendations described in International Commission on Illumination (CIE) CIE S 026/E:2018 [2].

Table A.1 Further photometric measures of the lighting system DS while driving (DS^+) or during the bright light application (DS^{++})

Photometric measures	DS^{++}	DS^{+}
Illuminance	550 lx	400 lx
S-cone-opic	1,664	1,210
M-cone-opic	735	534
L-cone-opic	615	447
Rhodopic	1,215	884
Melanopic	1,511	1,099

A.3 Description of the sham condition

The placebo intervention comprised a placebo UV light exposure delivered via DS (Fig. A.1, a). Participants were told that the DS system is operating in a “non-visual domain”. An “active” status of the intervention was indicated by a red signal lamp in the cockpit without emitting any supplementary visual or non-visual light.

Another element of the placebo condition was an alleged“air refreshing system” (AIR) contained in a portable box (40 × 30 × 50 cm; see Fig. A.3) positioned behind the co-driver’s seat. Participants were told that the AIR system was supposed to ionize and scent the air within the cabin. In regular intervals, the system circulated the air in the cabin and produced a humming noise.

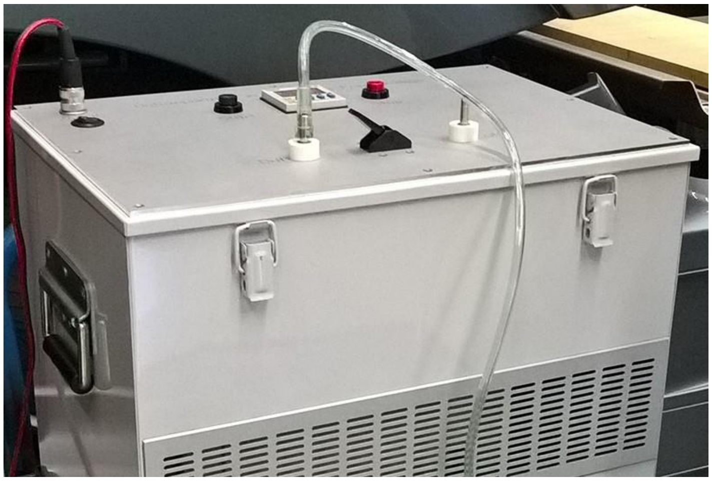

Figure A.3 The alleged air refreshing system of the placebo intervention (AIR); the box was positioned between the driver and the co-driver’s seat.

Appendix B: Study measures and assessments

B.1 Overview of assessment measures

Subjective and objective measurements used in the present study are summarized in Table B.1.

Table B.1 Measurements used in the study, along with their assessment levels and the dimensions the measurements served to assess

Measurements	***Measures***	***Dimension***
**SCREENING MEASUREMENTS**		
Pittsburgh Sleep Quality Index (PSQI) [3,4]	General sleep quality	Sleep quality
Morningness–Eveningness Questionnaire (D-MEQ) [5,6]	Chronobiological type	Chronotype
Epworth Sleepiness Scale (ESS) [7,8]	Sleep propensity during the day	Daytime sleepiness
**SUBJECTIVE MEASUREMENTS**		
Self-Assessment Scale for Sleeping and Awakening Quality (SSA) [9]	Sleep quality; awakening quality; somatic complaints; overall score	Subjective sleep quality
Karolinska Sleepiness Scale (KSS) [10]	Level of subjective sleepiness	Subjective alertness
Sleep diary*	Morning and evening protocols	Sleep characteristics
Driving protocol*	Weather conditions; special and technical incidents	Driving characteristics
Comfort ratings &<br>Ratings on visual side-effect	Acceptance with DS/light interventions and placebo conditions	Acceptance<br>Negative visual effects
**OBJECTIVE MEASUREMENTS – PSYCHOMETRIC/PERFORMANCE**		
Psychomotor Vigilance Task (PVT) [11]	Lapses; mean reaction time; fastest & slowest 10% of reactions	Alertness/ Sustained Attention
**OBJECTIVE MEASUREMENTS – PHYSIOLOGICAL**		
Wake Electroencephalography (EEG)	alpha spindles while driving	Drowsiness
Polysomnography (PSG)	Total sleep time; sleep efficiency; arousal index; sleep onset latency; sleep stages N1, N2, N3 and REM	Sleep quality
Saliva samples	Melatonin levels (pg/mL)	Melatonin release

* measurements marked with an asterisk were not quantitatively analyzed but served for documentation of unusual events or conditions. For measurement references see reference list B.8

All subjective measurements were questionnaires. Objective measurements included both psychometric, physiological measurements, physical light measurements and measurements of participants. All data were assessed and analyzed in a pseudo-anonymized manner.

B.2 Screening instruments

- The **Pittsburgh Sleep Quality Index** (PSQI) [[3]] is a self-rated questionnaire that assesses sleep quality and disturbances over a 1-month time interval. The PSQI contains 19 items, from which seven sleep component scores can be derived (e.g., subjective sleep quality, sleep latency). A global sleep quality score can be calculated as the sum of these component scores with a minimum of 0 and a maximum of 21. This score can be used to divide participants into *good* (score of 5 or lower) and *bad* sleepers (score above 5). Measures of consistency and validity are acceptable for the PSQI. In the present study, a validated German version of the PSQI was used [[4]].
- The **Morningness–Eveningness Questionnaire** (MEQ) [[5]] is self-assessment questionnaire to classify a person's chronobiological type based on questions regarding performance, sleep behavior, and well-being within a 24-hour time frame. The MEQ contains 19 items that are summed to form a composite score ranging from 16 to 86 indicating *definite morning type* (score: 70–86), *moderate morning type* (score: 59–69), *intermediate type* (score: 42–58), *moderate evening type* (score: 31–41), or *definite evening type* (score: 16–30). In the present study, the German version of the MEQ, called the **D-MEQ**, was used [[6]]. Internal consistency of the composite score is .82.
- The **Epworth Sleepiness Scale** (ESS) [[7]] is a self-administered questionnaire on subjective daytime sleepiness. The ESS contains eight questions about the probability of falling asleep (i.e., subjective sleep propensity) while engaged in different daily activities. A global score is derived from adding the answers together (coded from 0–3; the global score ranges from 0–24). Studies have shown sufficient consistency (Cronbach's alpha varies between 0.73 and 0.90) and test–retest reliability (the intraclass correlation coefficient ranges between 0.81 and 0.93). Participants in this study filled in the German version of the ESS [[8]]. According to a German validation study, scores higher than 10 categorize a participant as “clinically suspicious”, while

scores higher than 12 define a participants’ daytime sleepiness as “clinically relevant” [8].

B.3 Subjective Measurements

- The **Self-Assessment-Scale for Sleeping and Awakening Quality** (SSA) [9] is a morning questionnaire to measures sleep quality, awakening quality, and somatic complaints of the night before. In addition, an overall score of sleep and awakening quality can be derived. The SSA contains 20 items, the responses of which add up to the overall score with a minimum of 20 and a maximum of 80. Scores closer to 20 indicate a high quality of sleeping and awakening, whereas scores closer to 80 indicate a low quality of sleeping and awakening. The SSA was filled in during the morning before test drives.
- The **Karolinska Sleepiness Scale** (KSS) [10] quantifies the current subjective state of sleepiness on a 9-point rating scale ranging from 1 (*extremely alert*) to 9 (*very sleepy, great effort to keep awake, fighting sleep*). The KSS was presented on a computer screen and automatically time-logged.
- A **Sleep Diary** asked for sleep quality the night before. Participants recorded three sleep parameters, “time going to bed”, “time leaving bed”, and “sleep duration” for the previous night before the test drive.
- A **Driving Protocol** was used to control for further confounding parameters while driving. Participants rated two items after each test drive: (1) the primary weather conditions (with three categories: “sunny to 50% cloudy, no precipitation”, “partly cloudy (>50%) and possibly short rain- or snowfall”, and “cloudy and extensive rain- or snowfall”) and (2) leaving the car during the break (with two categories: “yes”, “no”) accompanied by disconnecting the EEG equipment.
- **Comfort Ratings** were used to compare the acceptance levels of the interventions. We used a 5-point semantic differential with the following four items: *pleasant-unpleasant*, *relaxing-activating*, *familiar-unfamiliar*, and *unobtrusive-obtrusive*. Additionally, visual side effects of the light intervention were evaluated on a 5-point scale (1 = *fully disagree*; 2 = *rather disagree*; 3 = *neither/nor*; 4 = *rather agree;* 5 = *fully agree*). The side effects questionnaire comprised five items: "The lighting system generated

reflections in the truck windows", "The lighting system produced glare", "The lighting system negatively affected my view", "The lighting system caused eye irritations" and "The lighting system distracted me".

B.4 Objective Measures—Psychometric/Performance

- The **PC Psychomotor Vigilance Task (PVT)** [11] is a reaction time (RT) test that measures several reaction parameters to a series of visual stimuli presented on a computer screen. Although the tasks last only 10 minutes, the test assesses alertness and sustained attention within a high stimulus-rate paradigm that is very sensitive to sleep loss and states of sleepiness [12]. The PVT stimulus consists of a red 4-digit number on a black screen counting upward from zero in ms. The inter stimulus interval had a random duration of 2 to 10 s. Participants were instructed to react as quickly as possible with a left mouse click (Logitech G 402-Hyperion-fury Gaming Mouse, sample rate 1000 Hz) as soon as the digits appeared. Participants immediately received visual feedback about their reaction time after each reaction. In the present study, reaction speed (i.e., reciprocal reaction time [RRT, 1/s]) and number of lapses (RT > 500 ms) were used for analysis. In general, participants took part in the PVT four times per study day, twice before (8:15 a.m., 9:45 a.m.) and twice after the test drives (2:00 p.m., 3:15 p.m.). Additionally, the PVT was presented once during the evening at 10:10 p.m. on Mondays and Fridays.

B.5 Objective Measures—Physiological

- **Wake Electroencephalography (Wake EEG)** was continuously recorded in real time during the daily test and driving phases from approximately 8:30 a.m. until 4:00 p.m. using ***BrainAmp***™ hardware and software (Brain Products GmbH, Munich, Germany). A set of 16 electrodes was positioned according to the International 10–20 system (Fig. B.1). EEG signals were recorded relative to the Cz electrode, and data were digitized at 250 Hz. All wake EEG data were stored on board for each half of the test drives (outward and return journey) and for each diurnal test phase.

In a driving context, alpha spindle rates (7–13 Hz) are a reliable marker to identify levels of fatigue and drowsiness [13]. The wake EEG analysis method of Simon et al. is based on a time–frequency decomposition of EEG data that automatically allows the derivation of several alpha spindle parameters (spindle rate, spindle duration, amplitude) under noisy recording conditions over pre-defined time periods [13]. In the present study, we applied this analysis method and used the most sensitive parameter [14,15], the alpha spindle rate, to quantify neurocognitive arousal levels in non-overlapping time periods of 6 minutes while driving under monotonous conditions. We refer to the publication of Simon et al. for further details regarding this analysis method [13]. The quality of the recorded EEG data was often insufficient, as data were occasionally either missing (electrodes lost contact during the 4-hour driving periods) or noisy (due to head movements and eye blinks).

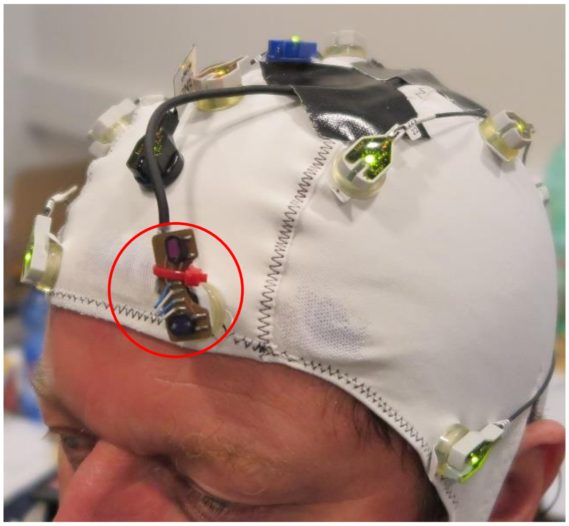

Figure B.1 Participant with the wake Electroencephalography (Wake EEG) cap; the light sensor 'LuxBlick 2.0' (indicated by ○) was mounted close to the forehead to evaluate vertical light exposure at eye level.

- **Polysomnography (**PSG**)** is the physiological “gold standard” to record sleep parameters to evaluate both the quality and quantity of sleep. PSG recordings depict measures like total sleep time, percentage of sleep period time (SPT) spent in different sleep stages, oxygen levels, and movement. PSG measurements were recorded on an on-board laptop on Mondays and Fridays (as well as on the first Sunday for diagnostic purposes) according to the standards of the American Academy of Sleep Medicine [16]. For the recordings, the digital PSG system NIHON KOHDEN EEG 1200 with the

recording software Polysmith 9.0 (Nihon Kohden, Tokyo, Japan) was used. The placement of electrodes was conducted as recommended by the American Academy of Sleep Medicine [[16]]. A PSG recording includes various physiological measurements, which are listed in Table B.1. PSG nights starting on Sunday included the full PSG for diagnostic purposes. Furthermore, this PSG was supposed to prevent first night effects of the following PSG test nights. During the actual experimental phase, a shortened PSG version, called “PSG light”, was administered on Monday and Friday nights to assess sleep quality. Scoring of sleep stages was mostly based on electrooculography, electromyography, and EEG. To evaluate objective sleep quality, standardized American Academy of Sleep Medicine outcome parameters were employed (e.g., total sleep time; sleep efficiency; arousal index; sleep onset latency; percentage of SPT spent in sleep stages N1, N2, N3, and REM). PSG recordings were not monitored using video. After applying all electrodes and initializing the system, the recordings were conducted after an impedance measurement and a biotest.

PSG measures assessed in the statistical analyses included the global sleep parameters total sleep time (TST in min) and sleep efficiency (%); latencies to sleep stages N1 and REM; sleeping stage distributions in percentage of SPT spent awake (% awake) and in sleep stages N1, N2, N3, and REM; and sleep interruptions, as measured by the arousal index (/hour), wake time after sleep onset (min), and changes of sleep stages. Statistical analyses of the PSG data included 15 recordings of nights during the experimental condition and 12 recordings of nights during the placebo condition.

Table B.2 Measurements of the first diagnostic polysomnography (PSG), along with their measures, channels, and electrodes

Measurement	***Measures***	***Number of channels***	***Electrodes***	***Reference electrode***
Electrooculography (EOG)	Horizontal and vertical eye movement	2	Pg1, Pg2	Fz
Electromyography (EMG)	Muscle tone at the chin	2	T1, T2, Pz	T2, Pz
	Leg movement	2	X1, X2	Gnd
Electroencephalography (EEG)	Brain activity	6	F3, F4, C3, C4, O1, O2	A1, A2
Nasal flow thermistor*	Nasal air flow	1	X5	Gnd
Abdominal belt*	Abdominal respiration	1	X7	Gnd
Snore microphone*	Snoring	1	X4	Gnd
Oxygen clip*	Pulse oximetry	1	Gnd	SpO2
Electrocardiography (ECG)*	Heart rate, blood pressure	3	T3, T4	CZ

* marked with an asterisk are measures that were not included in the cut-down version of the PSG, “PSG light”, conducted on Mondays and Fridays.

- **Melatonin levels** were analyzed in pg/mL using the non-extraction Melatonin direct Saliva enzyme-linked immunosorbent assay kit (RE54041; Tecan, Männedorf, Switzerland). During the evenings on Monday, Wednesday, and Friday, a total of 7 melatonin probes were collected via saliva samples (Blue Cap Salivette; Sarstedt AG & Co. KG, Nümbrecht, Germany) from each truck driver. Participants strictly followed the instructions to obtain salvia samples and refrained from eating, drinking, or smoking within thirty minutes before taking each sample. All samples were stored in a freezer at -20 °C until frozen shipment to a certified laboratory of the University of Regensburg.

For each saliva sample, a double determination of melatonin level was conducted and in case of inconsistent values, the sample was analyzed for a second time.
The parameter “Dim light melatonin onset” was not calculated, since light conditions during the evening test phases could not be as strictly controlled as in a laboratory setting to ensure constant dim light conditions below 40 lx or less.

B.6 Objective Measures—Physical

- The **light sensor “*LuxBlick 2.0*”** was used before, after, and during the test driving to continuously record vertical illuminances between 10 and 40000 lux close to the driver's eye level [[17]]. This device comprises a photosensor attached to the forehead of the driver (Fig. B.1) and measured photopic corneal illuminance levels with a sampling rate of 1 Hz. Data were forwarded to a battery-powered, body-worn data logger and stored on a USB pen-drive. Another LuxBlick sensor was mounted centrally on the windshield of the truck to log outdoor vertical illuminance as reference value. Technical details of the measurement device have been previously published [[17]].
- ***Fleetboard*™,** a built-in logistics system for trucks (Fleetboard by Daimler Truck AG, Leinfelden-Echterdingen, Germany), computer-logged driving data and analyzed various driving characteristics and vehicle parameters such as speed, fuel consumption, engine loads, usage of brakes and gas pedal, etc. The data were mainly used to monitor and control the drivers’ compliance to the study protocol (e.g., speed limit, use of Adaptive Cruise Control). Due to the extreme wintry driving conditions in Finland, the Fleetboard™ data could not be applied to reliably assess driving economy and efficiency.

B.7 Schematic overview of all applied assessments

Figure B.2 provides a depictive summary of the study protocol with all applied measures and assessments.

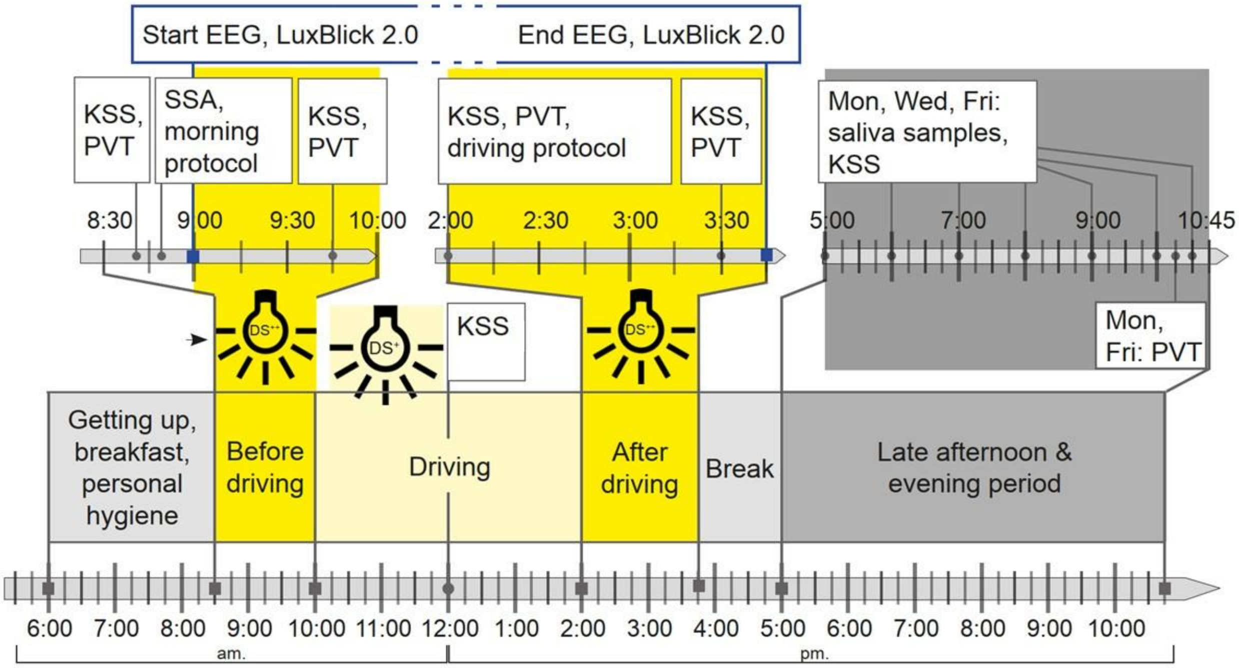

Figure B.2 Daily study protocol from Monday to Friday for both interventions. Lightbulbs indicate the two daylight supplement (DS) applications (the letters at their center specify the mode); DS and the placebo condition were continuously active from in-between before the drive in the morning until after the drive in the afternoon.

EEG Electroencephalography; KSS, Karolinska Sleepiness Scale; PVT, Psychomotor Vigilance Task; SSA, Self-Assessment Scale for Sleeping and Awakening Quality; Mon, Monday; Wed, Wednesday; Fri, Friday; DS^{++} = bright light exposure; DS^{+} = adaptive daylight supplementation.

DS^{++} was not active on Thursdays.

B.8 References

1. Canazei, M. *et al.* Feasibility and acute alerting effects of a daylight-supplementing in-vehicle lighting system – Results from two randomised controlled field studies during dawn and dusk. *Light. Res. Technol.* 1477153520982371 (2021) doi:10.1177/1477153520982371.
2. International Commission on Illumination (CIE). *CIE S 026/E:2018 CIE System for Metrology of Optical Radiation for ipRGC-Influenced Responses to Light*. https://cie.co.at/publications/cie-system-metrology-optical-radiation-iprgc-influenced-responses-light-0 (2018) doi:10.25039/S026.2018.
3. Buysse, D. J., Reynolds III., C. F., Monk, T. H., Berman, S. R. & Kupfer, D. J. The Pittsburgh Sleep Quality Index: a new instrument for psychiatric practice and research. *Psychiatry Res.* **28**, 193–213 (1989).
4. Backhaus, J., Junghanns, K., Broocks, A., Riemann, D. & Hohagen, F. Test–retest reliability and validity of the Pittsburgh Sleep Quality Index in primary insomnia. *J. Psychosom. Res.* **53**, 737–740 (2002).
5. Horne, J. A. & Östberg, O. A self-assessment questionnaire to determine morningness-eveningness in human circadian rhythms. *Int. J. Chronobiol.* **4**, 97–110 (1976).
6. Griefahn, B., Künemund, C., Bröde, P. & Mehnert, P. Zur Validität der deutschen Übersetzung des Morningness-Eveningness-Questionnaires von Horne und Östberg. *Somnologie* **5**, 71–80 (2001).
7. Johns, M. W. A New Method for Measuring Daytime Sleepiness: The Epworth Sleepiness Scale. *Sleep* **14**, 540–545 (1991).
8. Sauter, C. *et al.* Normative Values of the German Epworth Sleepiness Scale. *Somnologie - Schlafforschung Schlafmed.* **11**, 272–278 (2007).
9. Saletu, B., Wessely, P., Grünberger, J. & Schultes, M. Erste klinische Erfahrungen mit einem neuen schlafanstossenden Benzodiazepin, Cinolazepam, mittels eines Selbstbeurteilungsbogens für Schlaf- und Aufwachqualität (SSA). *Neuropsychiatrie* **1**, 169–176 (1987).
10. Åkerstedt, T. & Gillberg, M. Subjective and Objective Sleepiness in the Active Individual. *Int. J. Neurosci.* **52**, 29–37 (1990).
11. Khitrov, M. Y. *et al.* PC-PVT: a platform for psychomotor vigilance task testing, analysis,

and prediction. *Behav. Res. Methods* **46**, 140–147 (2014).

12. Basner, M. & Dinges, D. F. Maximizing sensitivity of the psychomotor vigilance test (PVT) to sleep loss. *Sleep* **34**, 581–591 (2011).
13. Simon, M. *et al.* EEG alpha spindle measures as indicators of driver fatigue under real traffic conditions. *Clin. Neurophysiol.* **122**, 1168–1178 (2011).
14. Sonnleitner, A., Simon, M., Kincses, W. E., Buchner, A. & Schrauf, M. Alpha spindles as neurophysiological correlates indicating attentional shift in a simulated driving task. *Int. J. Psychophysiol.* **83**, 110–118 (2012).
15. Sonnleitner, A. *et al.* EEG alpha spindles and prolonged brake reaction times during auditory distraction in an on-road driving study. *Accid. Anal. Prev.* **62**, 110–118 (2014).
16. American Academy of Sleep Medicine. *Das AASM-Manual zum Scoring von Schlaf und assoziierten Ereignissen: Regeln, Technologie und technische Spezifikationen*. (Steinkopff, 2012).
17. Hubalek, S., Zöschg, D. & Schierz, C. Ambulant recording of light for vision and non-visual biological effects. *Light. Res. Technol.* **38**, 314–321 (2006).

Appendix C: Additional results

C.1 Comfort ratings for the interventions—negative visual side effects

Study participants did not report any negative visual side effects (e.g., restrictions due to dazzling or glare) while driving in the DS or placebo conditions. The full distribution of the ratings is provided in Table C.1.

Table C.1 Reported frequencies for negative visual side effects for DS during driving compared to the inactive ultraviolet light exposure system

Ratings during driving	not at all	rather not	neither nor	rather	very
DS⁺ versus [placebo], '*The light system …*'					
'… generated reflections in the truck windows.'	7 [8]	1	0	0	0
'… produced glare.'	8 [8]	0	0	0	0
'…negatively affected my view.'	8 [8]	0	0	0	0
'… caused eye irritations.'	8 [8]	0	0	0	0
'… distracted me.'	8 [8]	0	0	0	0

C.2 Diagnostic polysomnography and screening results of participants

In the diagnostic polysomnography (PSG) conducted at the very beginning of the study, time in bed (TIB) was 424.2 ± 4.9 min, and the total sleep time (TST) of all truck drivers was on average 346.4 ± 14.9 min with a sleep efficiency of 81.8 ± 3.7%. In respect to sleep related breathing disorders, the Apnea–Hypopnea Index (AHI), Apnea Index (AI), and Oxygen Desaturation Index (ODI) were below the critical cut-off of < 10 events per hour of TST (i.e., AHI = 6.8 ± 2.9/h; AI = 4.0 ± 2.4/h; ODI = 7.1 ± 2.9/h).

In addition, there were no signs of clinically relevant motoric disorders with sleep disturbing effects due to bruxism or Periodic Limb Movements in Sleep (i.e. PLMS Index = 12.6 ± 7.6/h and PLMS Arousal Index = 4.4 ± 2.7/h).

The results of the screening questionnaires are listed in Table C.2. Regarding chronotype classification, three truck drivers were moderate morning types and five were intermediate or normal types. One truck driver (TD02) had a relatively high score of 12 in the Epworth Sleepiness Scale. Further exploration by a certified somnologist (RP) did not reveal any signs of a relevant hypersomnolence compromising fitness to drive or daytime functioning.

Table C.2 Participants' results in the applied screening questionnaires

A	***D-MEQ***	***ESS***	***PSQI***
	*Score (categorization)**	*Score (categorization)^*	*Score (categorization)°*
TD01	62 (mm)	8 (n)	4 (g)
TD02	54 (i)	12 (cs)	4 (g)
TD03	61 (mm)	7 (n)	4 (g)
TD04	58 (i)	9 (n)	4 (g)
TD05	57 (i)	10 (n)	5 (g)
TD06	56 (i)	10 (n)	3 (g)
TD07	50 (i)	6 (n)	5 (g)
TD08	64 (mm)	7 (n)	4 (g)
Mean±SD	**57.8±4.6**	**8.6±2.0**	**4.1±0.6**

*D-MEQ: German Morningness–Eveningness Questionnaire, categorization: i = intermediate type (42–58); mm = moderate morning type (59–69)
^ESS: Epworth Sleepiness Scale, categorization: n= normal (0–10), cs = clinically suspicious (11–12)
°PSQI: Pittsburgh Sleep Quality Index, categorization: g = good sleeper (< 6).

C.3. Polysomnography and objective sleep quality

To account for any positive or negative effects of the DS condition on sleep, we assessed sleep parameters derived from two polysomnography recordings (PSG light, see chapter B.5) at the beginning and the end of each study week (i.e., Monday and Friday night). Results are listed in Table C.3 for each experimental condition.

Table C.3. Comparison of sleep parameters between the active light intervention (DS) and the placebo condition based on polysomnography

	**DS** night 1 (MO)	**Placebo** night 1(MO)	**DS** night 2 (FR)	**Placebo** night 2 (FR)
**Measures**	*M (SD)*	*M (SD)*	*M (SD)*	*M (SD)*
included nights [32 recorded]	*n = 6*	*n = 8*	*n = 7*	*n = 6*
**GLOBAL SLEEP PARAMETERS**				
Total sleep time (min)	387.2 (13.5)	404.0 (8.5)	360.2 (42.2)	391.8 (31.8)
Sleep efficiency (%)	88.0 (5.1)	91.0 (2.5)	81.8 (8.7)	88.2 (4.6)
**SLEEP LATENCIES (MIN)**				
to sleep stage N1	21.8 (25.4)	12.8 (9.5)	15.1 (8.0)	16.2 (9.9)
to REM sleep	78.8 (60.0)	66.3 (15.7)	86.6 (70.1)	86.2 (33.8)
**SLEEPING STAGE DISTRIBUTION (%)**				
Awake	6.0 (5.3)	6.1 (3.1)	14.8 (9.9)	8.3 (4.9)
N1	6.5 (3.2)	10.2 (6.4)	9.0 (4.7)	13.8 (13.2)
N2	42.2 (10.8)	42.9 (8.7)	35.5 (12.3)	38.3 (8.9)
N3 (slow wave sleep)	28.2 (10.3)	22.4 (9.3)	25.4 (8.4)	24.5 (10.8)
REM sleep	17.2 (2.4)	18.4 (3.7)	15.2 (5.1)	15.3 (3.7)
**SLEEP INTERRUPTIONS**				
Arousal-Index (/hour TST)	13.2 (6.4)	14.9 (9.0)	15.1 (7.4)	20.3 (17.2)
Wake time after sleep onset (min)	25.5 (24.1)	26.7 (14.2)	62.4 (41.6)	34.9 (19.7)
Changes of sleep stages (/hour TST)	14.5 (4.8)	16.0 (15.2)	17.0 (4.5)	16.7 (7.0)

TST: Total Sleep Time
From 32 recorded nights, 27 nights were included in the analyses. One first (MO) DS night was excluded from analyses due to system failure; two second (FR) placebo nights were removed from analyses due to technical errors; one first (MO) DS night was removed due to strong back pain of the test driver throughout the night which led to premature termination of the night; and one second (FR) DS night was excluded due to strong winds throughout the night, which led to rocking motions of the truck cabin.

Appendix D: Unpublished pilot study

Farkas, Sophie-Christie. Daylight+. *The efficacy of the new lighting system in truck interiors to support the alertness of professional drivers.* (Daylight+. Die Wirksamkeit des neuen Beleuchtungssystems im Lkw-Innenbereich zur Unterstützung der Alertness von Berufskraftfahrer). Unpublished master's thesis; Innsbruck University; (2014).

D.1 Aim of the study and main results

A pilot study was conducted to investigate whether the use of biologically active light in the driver's cabin as a supplement to daylight in natural driving situations could improve the driver's alertness and vigilance and lead to more economical driving performance.

Methods: The sample consisted of eight healthy, experienced truck drivers from Daimler AG. The measurements were carried out on a subjective (Tiredness Symptom Scale, assessment of fitness; Karolinska Sleepiness Scale, assessment of driving performance), performance-related (Psychomotor Vigilance Task; Mackworth Clock Test, assessment of driving performance) and physiological (EEG) level. There were two lighting conditions for the test drives: an experimental condition with blue-enriched and biologically active light (460 nanometers; see Figure D.1, D.2 a) and a control condition with dimmed red light with no biological effect (Figure D.2 b). Each driver completed at least two test drives in the morning and two in the afternoon under each condition. The test results were compared in a within-participants design.

Results: The mean illuminance across all test drives was 1492.4 lx (SD = 351.3) for the active lighting condition and 367.0 lx (SD = 160.9) for the placebo condition. A comparison of the test results for the experimental and control conditions revealed significant differences in the Mackworth Clock Test. The truck drivers performed better after the experimental condition. The most striking result was seen in economic driving performance, which was much better under the biologically active light condition.

Discussion: This study was the first to demonstrate a significant effect of biologically active light on performance-related alertness and vigilance in a natural driving situation. In addition, it showed that more alert and attentive drivers drove more economically after exposure to bright and blue-enriched light. These results are of great importance in practice, as lower fuel consumption and less wear and tear on vehicles can lead to considerable monetary and environmental cost savings.

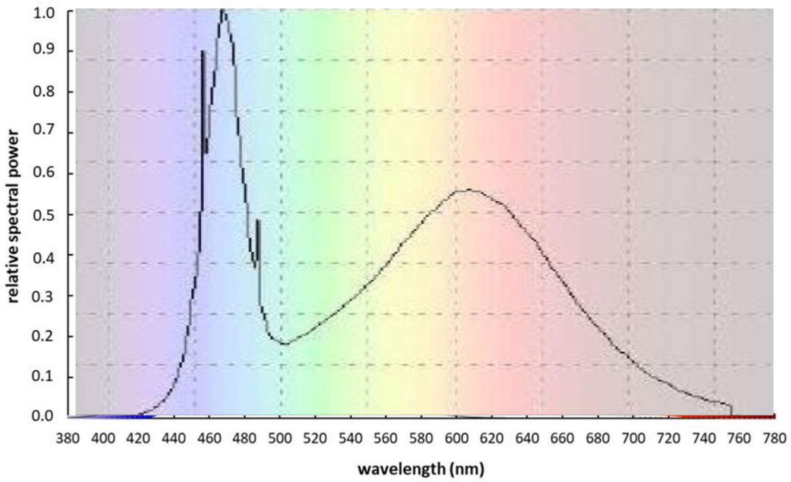

Figure D.1 Emission spectrum of the blue-enriched polychromatic light spectrum of the original DS lighting system.

a **b**

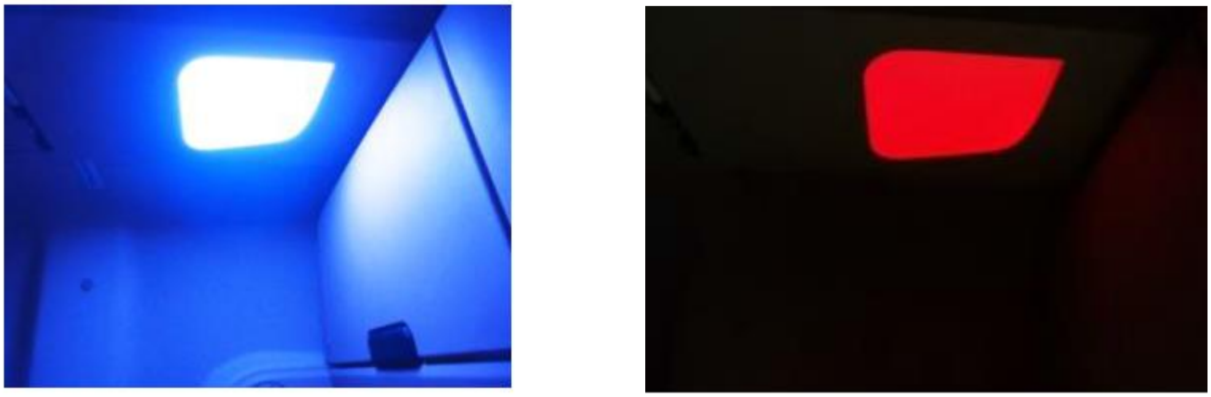

Figure D.2 The in-vehicle daylight-supplementing lighting system (DS+);
a: Active light condition with blue-light exposure above the driver`s seat; **b:** Placebo condition with dim red light (< 30 lx).
